# Transcriptomic profiles of susceptibility and resilience to stress in the amygdala and hippocampus of male rats

**DOI:** 10.1016/j.ynstr.2025.100754

**Published:** 2025-08-26

**Authors:** Kimberly L.P. Long, Sandra E. Muroy, Siamak K. Sorooshyari, Mee Jung Ko, Yanabah Jaques, Kishant Mohan, Peter Sudmant, Daniela Kaufer

**Affiliations:** a Helen Wills Neuroscience Institute, University of California, Berkeley, Berkeley, CA, 94720, USA; b Department of Integrative Biology, University of California, Berkeley, Berkeley, CA, 94720, USA; c Department of Bioengineering, University of California, Berkeley, Berkeley, CA, 94720, USA

**Keywords:** Stress, Hippocampus, Amygdala, RNA sequencing, Anxiety

## Abstract

Traumatic experiences elicit a wide range of cognitive responses in both humans and animals, leading to diverse outcomes such as enhanced performance, cognitive impairment, or the development of mood and anxiety disorders like posttraumatic stress disorder (PTSD). A key challenge in understanding these varied responses is to decipher the underlying biological mechanisms that contribute to individual variability in trauma resilience or susceptibility. The purpose of this study was to elucidate the molecular bases for these differences, focusing on the amygdala and hippocampus—brain regions integral to stress responses. We exposed adult, male rats to an acute, severe stressor and profiled persistent anxiety-like behavior outcomes 7 days later. We investigated the transcriptional signatures in the basolateral amygdala and hippocampal dentate gyrus via bulk RNA sequencing from animals with behavioral outcomes indicative of stress resilience or vulnerability. Our results suggest that the basolateral amygdala and dentate gyrus display distinct transcriptomic changes following acute, severe stress. Furthermore, we identified specific region-dependent genes related to insulin signaling, neural plasticity, and stress responses that correlate with resilient and vulnerable phenotypes. Notably, a larger number of genes separated stress-resilient animals from both control and stress-susceptible animals, underscoring that an active molecular response, particularly in the hippocampus, facilitates protection from the long-term consequences of severe stress. These findings provide novel insight into the mechanisms that engender individual variability in the behavioral responses to stress and offer new targets for the advancement of therapies for stress-induced neuropsychiatric disorders.

## Highlights

• Amygdala/hippocampus display distinct transcriptomic signatures after severe stress.
• Region-specific genes link insulin signaling, plasticity to susceptibility/resilience.
• Resilience is associated with active transcriptional responses in the hippocampus.
• Genomics offer insights into mechanisms driving individual variability after stress.

## Graphical abstract

**Figure:**
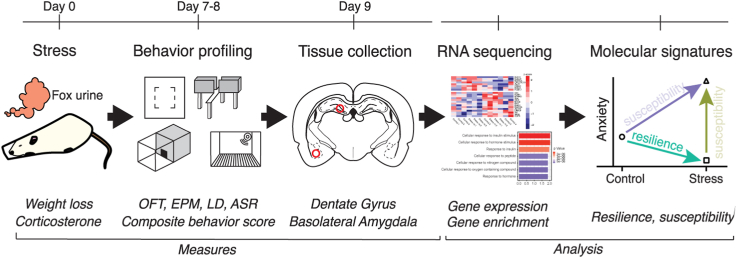

## 1 Introduction

Traumatic experiences bring about diverse responses in humans and animals, ranging along a continuous spectrum from enhanced to severely impaired cognitive function (Yehuda and LeDoux, 2007; Richter-Levin and Sandi, 2021). In particular, a subset of individuals who experience trauma fail to recover from the acute effects of stress and develop long-lasting mood and anxiety disorders, such as posttraumatic stress disorder (PTSD) (Kessler, 1995). Understanding the biological basis of individual variability driving the ability or failure to recover from trauma may aid in the development of new treatments to promote resilience; however, the neural and molecular mechanisms that bring about the various behavioral responses to trauma remain largely unknown.

Molecular changes to limbic structures are key to the physiological, affective, and behavioral responses to stress (Roozendaal et al., 2009; McEwen, 1999). Stress immediately alters gene expression in both the amygdala and hippocampus (von Ziegler et al., 2022; Lori et al., 2019; Gray et al., 2014). These transcriptional changes can be long-lasting, particularly following chronic or traumatic stress (Gray et al., 2014; Sillivan et al., 2019; Shen et al., 2019; Ponomarev et al., 2010). Molecular responses, in turn, can drive changes to the structure of cells and circuits. For example, both acute and chronic stressors alter the landscapes of the amygdala and hippocampus in a region-specific manner via the restructuring of dendritic spines and the growth or shrinkage of neurites (McEwen, 1999; McEwen et al., 2016; Vyas et al., 2002; Mitra et al., 2005; Chen et al., 2008; Galea et al., 1997).

Ultimately, persistent changes to molecular programs after stress may alter the structure and function of limbic circuitry and contribute to anxiety-like behavior (McEwen et al., 2016; Vyas et al., 2002; Mitra et al., 2005; Fa et al., 2014; Datson et al., 2013; Kirby et al., 2013). Hence, individual variability in molecular responses to stress may be a driver or a correlate of an individual's susceptibility or resilience to mood and anxiety disorders after trauma (Shen et al., 2019; Sillivan et al., 2017; Nasca et al., 2019; Daskalakis et al., 2014). However, few studies have investigated the delayed molecular correlates of individual variability in behavioral outcomes following acute, severe stress, and the mechanisms and specific genes underlying individual behavioral responses to trauma remain poorly defined. Our previous work found that patterns of oligodendrocyte and myelin density in the hippocampal dentate gyrus and basolateral amygdala correlate with and contribute to resilience and susceptibility to anxiety-like behavior in a rat model of acute trauma (Long et al., 2021). Here, we hypothesized that transcriptional signatures in these regions correlate with divergent behavioral outcomes after an acute stressor. Using a single, severe stressor and behavioral profiling in male rats, we identified region-specific gene expression patterns—particularly related to insulin signaling, plasticity, and stress—that distinguish resilient and vulnerable animals, highlighting potential molecular targets for regulating trauma outcomes.

## 2 Materials and methods

### 2.1 Study design

The aim of this study was to determine amygdala and hippocampal transcriptomic profiles of resilience and susceptibility to trauma. Male rats were exposed to an acute, severe stressor, and outcomes were assessed using physiological (serum corticosterone), behavioral (avoidance and startle assays), and transcriptional (RNA sequencing) measures. The experimental designs and outcome measures were chosen prior to initiating each experiment. For all experiments, animals were randomly assigned to experimental groups. Investigators were blind to stress conditions during behavioral assays and data quantification. The criteria for animal exclusion were experimental error and poor RNA quality (detailed below). Sample sizes and statistics for each experiment are provided in figure legends.

### 2.2 Animals

All animal care and procedures were approved by the UC Berkeley Institutional Animal Care and Use Committee. A total of 40 adult (post-natal day 65) male Sprague Dawley rats were purchased from Charles River and pair-housed on a 12:12 light-dark cycle (lights on at 0700 h) in our facility at the University of California, Berkeley. Only male rats were used in this experiment due to previous findings from our lab that female Sprague Dawley rats exposed to this stress paradigm do not develop the spectrum of low to high avoidance and startle behavior seen in males (unpublished data). Rats had *ad libitum* access to food and water and were given one week to acclimate to the facility before testing began. All rats underwent gentle handling (being picked up and held for about 2 min) for 5 days prior to stress.

### 2.3 Stress

To study acute, severe trauma exposure, we coupled a single exposure to predator scent (fox urine) with 3 h of immobilization. Rats were randomly assigned to undergo stress or remain in the home cage. Rats were run in pairs in cohorts of 10 animals each, with numbers of control and stress animals counterbalanced between cohorts. In the stress group (n = 20), rats underwent acute immobilization stress with exposure to predator scent. Rats were restrained in Decapicone bags (Braintree Scientific, Braintree, MA), and cage mates were placed side-by-side in an empty holding cage inside a fume hood from 0900 to 1200 h. A cotton ball infused with 1 mL of fox urine (Trap Shack Co. Red Fox Urine, amazon.com) was placed in the center of the holding cage, and a lid was placed on the cage. Blood sampling occurred throughout stress (detailed below). After cessation of stress, animals were released into a clean cage to allow for self-grooming. After 1 h, rats were returned to a clean home cage. Rats in the control group (n = 20) received a cage change at the same time of day. To prevent transfer of stress and fox odors to control animals in the colony room, stress-exposed animals were housed in a separate room for 2 nights after stress before being returned to the main colony. It should be noted that housing the stress-exposed animals separately for two days after stress may have added some additional environmental stress. However, we felt that the tradeoff of temporarily housing the animals separately was lesser than that of permanently treating the control and stress groups differently by always housing them in separate rooms.

### 2.4 Weights

Animals were weighed on days −3 (handling), 0, +1, +2, +3, +7 (after behavior profiling day 1), +8 (after behavior profiling day 2), and +9 (day of sacrifice) relative to stress. One cohort of animals was mistakenly not weighed on the day of and day after stress; hence, these values are absent from plots of weight loss. Percent weight loss after stress was calculated as 100∗(weight_Day +1_ - weight_Day 0_)/weight_Day 0_.

### 2.5 Serum corticosterone sampling

To quantify hormonal stress responses, tail vein blood was collected from each rat for corticosterone sampling at 0 min, 30 min, and 3 h into acute immobilization stress. Blood samples were centrifuged at 9391 g for 20 min at 4 °C, and serum was extracted and stored at −80 °C. Samples from the 30-min and 3-h time points were assayed using a Corticosterone EIA kit (Arbor Assays, Ann Arbor, MI).

### 2.6 Behavioral battery

Animals underwent a behavioral battery 7 days after stress exposure to characterize the extent of persistent behavioral changes. This design was modeled from previous studies in rats to identify animals with persistent changes to fear, startle, and anxiety-like behavior after severe stress (Daskalakis et al., 2014; Cohen and Zohar, 2004; Cohen et al., 2004, 2006). The 7 day time period was originally chosen to model human post-traumatic stress disorder, which is characterized by changes to mood and anxiety behavior that persist for at least one month. All behaviors were conducted between 0800 and 1400 h. Prior to all tests, animals were brought to the testing space and allowed at least 30 min to acclimate. One day prior to stress, all animals went through a 5 min baseline open field test (OFT) under dim lighting (15 lux). Seven days after acute immobilization stress, all rats were individually profiled for anxiety-like behaviors using 6 different behavioral tests: OFT in a brightly lit environment (OFT Light), OFT in a dimly lit environment (OFT Dim), light/dark box (LD), elevated plus maze (EPM) under bright white light, EPM under dim red light, and acoustic startle response (ASR). These behavior assays were chosen to reflect and model human behavioral diagnostic clusters for PTSD, which include avoidance (OFT, EPM, LD) and hyperarousal or non-habituation to acoustic stimuli (ASR)(Cohen and Zohar, 2004). Low light versions of the OFT and EPM were utilized as low anxiogenic versions of their full light counterparts, allowing for further characterization of exploratory and anxiety-like behavior. The behavioral battery spanned two days with the following sequence: Day 1 – OFT Light, EPM Light, ASR; Day 2 – OFT Dim, LD, EPM Dim. The order of assays was not randomized and was consistent across all animals. While this battery may risk test-induced reactivation of stress responses, our approach is specifically designed to identify robust, trait-like anxiety rather than a transient state response to a single test. The idea is that a truly anxious phenotype will manifest as consistent avoidance behavior across a variety of novel, mildly anxiogenic contexts. Importantly, in assays of approach-avoidance conflict and anxiety-like behavior, such as the elevated plus maze, the novelty of the context is a critical driver of approach-avoidance conflict. For this reason, repeating a single assay is known to influence subsequent behavior and is not a viable strategy. Our use of a battery of different tests allowed us to characterize anxiety-like behavior more accurately while maintaining novelty for each distinct assessment. After placing an animal into an arena, the experimenter exited the room. Animals were given 10 min of rest in the home cage in between each test. One control animal was excluded from subsequent analyses due to experimental error during behavior, in which the animal was not given time to habituate to the room prior to behavior testing.

### 2.7 Open field test (OFT)

Each rat was placed in an unenclosed plastic box (50 l x 50 w x 58 h in cm) and was given 10 min to freely explore the arena. All animals were placed along a wall at the beginning of the test. Behavior was recorded with cameras positioned above the arena and connected to GeoVision software (GeoVision Inc., Taiwan). Behavior was scored as latency to, frequency, and total amount of time spent in the center of the box (designated by a 25 × 25 cm square), as well as total distance traveled in the arena and in the center using EthoVision software (Noldus, Leesburg, VA). The OFT Light was conducted under full lighting (280 lux). The OFT Dim was conducted in a different but identically structured box in the same room under 15 lux. The arena was cleaned with 1 % acetic acid followed by Formula 409 All Purpose Cleaner after each animal.

### 2.8 Elevated plus maze (EPM)

Rats were allowed to explore an EPM for 10 min (arm dimensions: 10 w x 60 l in cm; closed arm enclosed by walls 51 cm in height; apparatus elevated 50 cm off the ground). The arms of the EPM were 10 cm wide. Behavior was recorded by a JVC Everio camera (JVCKENWOOD, Tokyo, Japan) mounted above the apparatus. The criteria for open arm exploration was considered as more than half of the body (and both forepaws) placed into the open arm. Latency to and total time spent in the exposed open arms, as well as time spent in the protected closed arms, were quantified by observers blind to condition. The EPM Light was conducted at 240 lux, while the EPM Dim was conducted under dim red light. The apparatus was cleaned with 1 % Process NPD Disinfectant (STERIS Life Sciences) after each animal.

### 2.9 Light-dark box (LD)

Each rat was placed in a structure consisting of an enclosed dark box separated by a divider with a small door leading to an unenclosed light box (each box 15 w x 15 l × 8 h in inches). All animals were placed into the dark half of the box and given 10 min to explore. Behavior was recorded by a JVC Everio camera (JVCKENWOOD, Tokyo, Japan) mounted above the apparatus. Measures for distance traveled as well as latency to, frequency, and total time spent in the exposed side were quantified by observers blind to condition. The arena was cleaned with 70 % ethanol after each animal.

### 2.10 Acoustic startle response (ASR)

Each rat was placed into an isolated Coulbourn sound-attenuating fear conditioning chamber (12 w x 10 l × 12 h in inches) and was exposed to 5 min of background noise (∼55 dB). This was followed by 70–110 dB white noise pulses lasting 10 ms, with an inter-stimulus interval of 15–30 s. All tones were calibrated with a handheld decibel meter each day prior to testing. Behavior was recorded over two different trials: habituation (110 dB tones presented 15 times to assess initial responses and subsequent habituation) and threshold determination (70–110 dB tones presented in pseudo-random order, with each tone played 5 times in total). Behavior was recorded using a Coulbourn Instruments camera connected to a computer with FreezeFrame software (Coulbourn Instruments, Whitehall, PA). The boxes were cleaned with 70 % ethanol after each animal. Fear and startle behavior were assessed by Ethovision software analysis of activity change (measured as percent pixel change from frame to frame). The amplitude of startle was quantified as the maximum activity minus baseline activity in the 50 ms surrounding the startle pulse. Mean startle response was calculated as the mean of all startle amplitude scores across all 15 stimuli from the habituation phase. Sensitization was calculated as 100∗[(mean startle amplitude to stimuli 13–15)-(mean startle amplitude to stimuli 1–3)]/(mean startle amplitude to stimuli 1–3).

### 2.11 Composite scoring

To standardize and quantify behavior across multiple behavior tests, we adapted the method of Cutoff Behavioral Criteria developed by Cohen and Zohar (2004), expanded by Richter-Levin and colleagues (Richter-Levin et al., 2019; Ardi et al., 2016; Ritov et al., 2016), and described in Long et al., (2021). For each measure from the avoidance tests (OFTs, EPMs, LD) a behavioral cutoff threshold was defined by the 20th percentile of the control distribution. For measures in which greater scores indicate greater anxiety-like behavior (latency to the anxiogenic zone, time spent in an anxiolytic zone), the 80th percentile of the control group was used. Binary scoring was applied: Animals falling outside the threshold were marked as “affected” and received a score of 1 for that measure. Scores were then summed across all tests (minimum 0, maximum 20). High scores represent consistent anxiety-like behavior across all tests. Animals with high scores outside the range of the control group were considered “stress vulnerable,” while those within the range of control animals were considered “stress resilient.”

### 2.12 Fresh tissue collection and RNA sequencing

On day 9 after stress, animals were anesthetized with isoflurane and rapidly decapitated. Brains were extracted, flash frozen by floating on liquid nitrogen, and stored at −80 °C. A subset of 12 animals was selected for RNA sequencing based on composite anxiety-like behavior scores: 3 stress-exposed animals with low scores (0–2), 3 stress-exposed animals with high scores (13–15), and 6 control animals from across the spectrum (0–6, average = 3.67). Sections of 200 μm thickness were collected under sterile conditions on a Leica cryostat, and punches of 0.75 mm diameter were taken from the dorsal dentate gyrus and basolateral amygdala (rat AP coordinates −2.9 to −5.28 relative to Bregma) using a Rapid-Core Sampling Tool (Electron Microscopy Sciences, Hatfield, PA). Punches were separated by hemisphere, and 7–9 punches were collected per hemisphere. Samples were stored at −80 °C until processing. Tissue punches from the left hemisphere were homogenized in TRIzol reagent (Invitrogen, Waltham, MA), and RNA was extracted and treated with DNase (DNase I, RNase-free, New England Biolabs, Ipswich, MA) according to the TRIzol reagent user guide (Pub. No. MAN0001271, Rev. A.0). The left hemisphere was chosen for the purposes of standardization and consistency across animals. For RNA sequencing, all postprocessing (including cDNA library preparation) and sequencing was performed by the Vincent J. Coates Genomics Sequencing Laboratory at UC Berkeley. RNA quality scores were determined for each sample on an Agilent 2100 Bioanalyzer (Agilent Technologies, Santa Clara, CA). One sample (from a control animal with composite behavior score of 0) had low RNA quality, and RNA was re-extracted but later excluded from analysis (see Statistical Analysis section). RNA was poly-A selected, and library preparation was conducted on a Biomek FXp with Kapa Biosystems reagents. Sequencing was performed on the Illumina HiSeq4000 (Illumina, San Diego, CA).

Reads were aligned to the *Rattus norvegicus* Rn5 genome assembly using Spliced Transcripts Alignment to a Reference aligner (STAR/2.6.0a) (Dobin et al., 2013) with gene annotations provided from the ensemble build 75. Transcript/gene level quantification and differential gene expression were performed using rsem/1.3.1 + ebseq (Li and Dewey, 2011; Leng et al., 2013). Gene Set Enrichment Analysis (GSEA) (Mootha et al., 2003; Subramanian et al., 2005) was performed on sets of differentially expressed genes (FDR <0.05) to determine enriched biological themes.

### 2.13 Statistical Analysis

For behavior, weight, and corticosterone data: All bars and error bars are presented as mean ± standard error of the mean (SEM). For continuous data, two sample comparisons were performed by two-tailed student's *t*-test. Normality of the residuals was assessed via the D'Agostino-Pearson omnibus test. If the assumption of normality failed, data were log transformed, and a student's *t*-test performed. If the assumption of normality failed again, a non-parametric Mann Whitney test was performed on the untransformed data. If the assumption of homogeneity of variance was violated, as determined by the F test, a Welch's *t*-test was performed. For count data, control and stress-exposed groups were compared via a generalized linear model using a Poisson distribution. To compare the relationships between behavioral and/or transcriptional measures, we computed Pearson correlations. For the acoustic startle paradigm measures of habituation over time and startle threshold, we conducted a two-way repeated measures ANOVA. In all tests, the alpha value was set at 0.05. Analyses were performed using IBM SPSS 19 (SPSS, Inc., Chicago, IL), GraphPad Prism version 9.1.1 for Mac OS X (GraphPad Software, San Diego, California USA, www.graphpad.com), R version 4.4.2 (2024-10-31), or Matlab version R2022b (Mathworks, Natick, MA). Hierarchical clustering of behavior was performed with the Seaborn package in Python (Version 3.7.1, Python Software Foundation, Wilmington, DE). All statistical analyses related to behavior, corticosterone, and correlations to sequencing data can be found in supplemental file S1 “Statistics.”

For RNA-seq data: the R EBSeq (Leng et al., 2013) package's posterior probability of being differentially expressed (PPDE >0.95, FDR <0.05) was used to determine statistical significance. One animal was excluded from all analyses due to experimental error during behavior assays (see Behavioral battery section above), and one hippocampal RNA sample was excluded from analyses due to contamination. Specifically, the sample was flagged for low RNA quality and was subsequently re-extracted from the remaining homogenized tissue. Although the re-extracted sample had a passing RNA integrity number, principal component analysis revealed that the sample did not cluster with any other samples (Fig. S1), suggesting that re-extraction compromised RNA integrity or introduced contamination. As a result, this sample was excluded from further analyses. Similarly, one amygdala sample was excluded from analysis as it did not cluster in PC space with any other samples. To correlate *Cartpt* expression values to behavior scores, transcripts per million (TPM) values for *Cartpt* were transformed as log_2_(TPM+1) to achieve normally distributed values. Normality of this distribution was tested using the Shapiro-Wilk Test and the D'Agostino-Pearson Test. The data did not significantly deviate from normality.

## 3 Results

### 3.1 Acute physiological responses to a severe stressor correlate with subsequent resilience and vulnerability to anxiety-like behavior

**Fig. 1:**
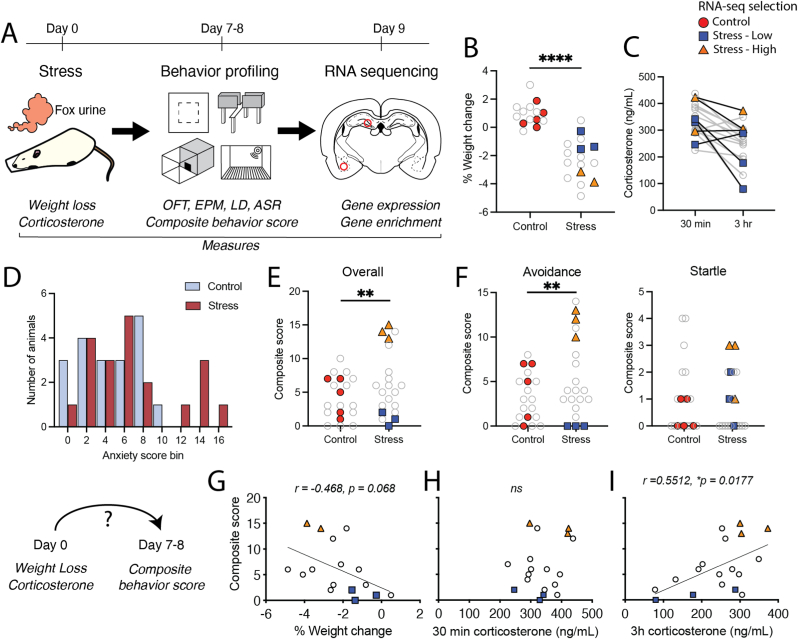
Experimental design and selection of control, stress-susceptible, and stress-resilient rats. A. Experimental design. Male Sprague-Dawley rats were subjected to a severe stressor (3 h of immobilization with exposure to fox urine). Body weight was measured on the day of and day after stress, and serum samples were collected for corticosterone measurements at 30 min and 3 h into stress. Behavior profiling occurred on days 7 and 8 after stress, and whole brains were flash frozen on liquid nitrogen on day 9 for tissue punch collections and bulk RNA sequencing from the left dorsal dentate gyrus and basolateral amygdala (red circles). B. Percent changes in body weight from day 0 (day of stress) to day 1. Negative values indicate weight loss (2-tailed unpaired *t*-test with Welch's correction for unequal variance, t(24.38) = 7.466, p < 0.0001). A set of 5 control and 4 stress-exposed animals, including one animal in the stress-high group, is not shown due to missing weight information (see Methods). C. Serum corticosterone measurements at 30 min and 3 h into stress exposure. D. Histogram of composite anxiety-like behavior scores (bin size = 2). E. Distribution of individual composite behavior scores from control and stress-exposed rats (Poisson generalized linear model, coefficient 0.41087±0.14274, p = 0.004). Highlighted points indicate the individual animals selected for RNA sequencing. F. Sub-scores of composite behavior derived from avoidance (left, Poisson generalized linear model, coefficient 0.26360±0.08064, p = 0.001) and startle (right, Poisson generalized linear model, coefficient −0.05129±0.31623, p = 0.87) assays. G. Pearson correlation of percent body weight change to composite behavior scores (r = −0.468, p = 0.068). H. Correlation of corticosterone at 30 min to composite behavior scores (r = 0.2443, p = 0.3287). I. Correlation of corticosterone at 3 h to composite behavior scores (r = 0.5512, p = 0.0177). OFT, open field test. EPM, elevated plus maze. LD, light-dark box. ASR, acoustic startle response. ∗p < 0.05, ∗∗p < 0.01, ∗∗∗∗p < 0.0001.

We sought to characterize the molecular signatures of resilience or vulnerability to the effects of acute, severe stress in male rats. We employed a controlled experiment that paired acute immobilization with predator scent stress followed by multi-assay characterization of anxiety-like behavior. We subjected 20 adult, male Sprague-Dawley rats to 3 h of acute immobilization with simultaneous predator scent stress (fox urine; Fig. 1A). Another 19 animals served as controls and remained minimally disturbed in the home cage. Predator odor is widely used in the field of stress neurobiology as an ethologically relevant stressor in mice and rats (Daskalakis et al., 2014; Santi et al., 2018; Koolhaas et al., 2011). Pairing acute immobilization with predator odor thus models an inescapable, life-threatening stressor. Previous work from our lab demonstrated that this paradigm induces a range of avoidance behavior when examined 1 week after the stressor (Long et al., 2021). Only male rats were used in this experiment due to previous findings from our lab that female Sprague Dawley rats exposed to this stress paradigm do not develop the spectrum of low to high avoidance and startle behavior seen in males (unpublished data).

Stress-exposed rats lost significantly more weight by the day after stress than control animals (Fig. 1B). Stress-exposed rats also displayed high circulating corticosterone at both 30 min and 3 h into stress (Fig. 1C). Thus, as we have previously shown, this acute, combinatorial design is a severe stressor that elicits a potent physiological stress response in rats (Long et al., 2021; Muroy et al., 2016; Breton et al., 2021).

To identify subsets of animals displaying persistent high or low anxiety-like behavior after stress, all animals were given 7 days of recovery with minimal disturbance before undergoing a 2-day behavioral profiling battery. This period of time has been used and validated in previous studies in rats to identify animals with persistent changes to fear, startle, and anxiety-like behavior after severe stress (Daskalakis et al., 2014; Cohen and Zohar, 2004; Cohen et al., 2004, 2006). The behavioral battery consisted of multiple tests of approach-avoidance conflict and acoustic startle (Fig. 1A). Consistent with our previous work (Long et al., 2021), we found that stress-exposed rats displayed considerable individual variability in each behavioral metric (Fig. S2). Furthermore, patterns of significant correlations between measures within and across tests indicated that individual stress-exposed rats displayed consistent avoidance phenotypes across assays (Fig. S3). This replicates our previous work suggesting that exposure to this stressor yields consistent patterns of avoidance behavior in male rats (Long et al., 2021). We quantified these overall anxiety phenotypes by constructing composite behavior scores from individual metrics (Long et al., 2021; Cohen and Zohar, 2004), and we observed that control and stress-exposed animals displayed a range of anxiety phenotypes (Fig. 1D). Notably, a subset of stress-exposed rats displayed high scores that were driven largely by avoidance behavior (Fig. 1E and F), while others were indistinguishable from controls. This is consistent with the hypothesis that only a subset of individuals is vulnerable to the display of persistent anxiety-like behavior following a traumatic event (Richter-Levin et al., 2019; Ardi et al., 2016; Ritov et al., 2016).

Immediate physiological and endocrine responses to trauma may predict whether an individual later displays PTSD (Cohen et al., 2006; McFarlane et al., 1997; Shalev et al., 1998; Yehuda et al., 1998a, 1998b; Monari et al., 2024). We, therefore, tested whether physiological measures such as stress-induced weight loss and corticosterone release correlate with anxiety-like behavior in our rat model of acute trauma exposure. Weight changes trended towards a negative correlation with composite anxiety-like behavior scores (p = 0.068), such that animals that lost more weight by the day after stress tended to show greater anxiety-like behavior 7 days later (Fig. 1G). This was driven by a significant correlation of weight change and composite startle scores (Fig. S4A). Serum corticosterone measured at 30 min into the stress exposure was not correlated with behavioral scores (Fig. 1H; Fig. S4B). Strikingly, however, corticosterone at 3 h (the end of stress) was significantly, positively correlated with overall composite scores and avoidance sub-scores (Fig. 1I; Fig. S4C). This may suggest that sustained, high release of corticosterone during a stressor contributes to subsequent anxiety-like behavior, while maintaining lower levels of corticosterone contributes to resilience. These findings support the hypothesis that an individual's early response to stress, whether physiological, neural, and/or hormonal, corresponds with susceptibility to subsequent anxiety-like behavior (Yehuda et al., 1998b).

### 3.2 The basolateral amygdala and dentate gyrus display distinct transcriptomic changes following acute, severe stress

**Fig. 2:**
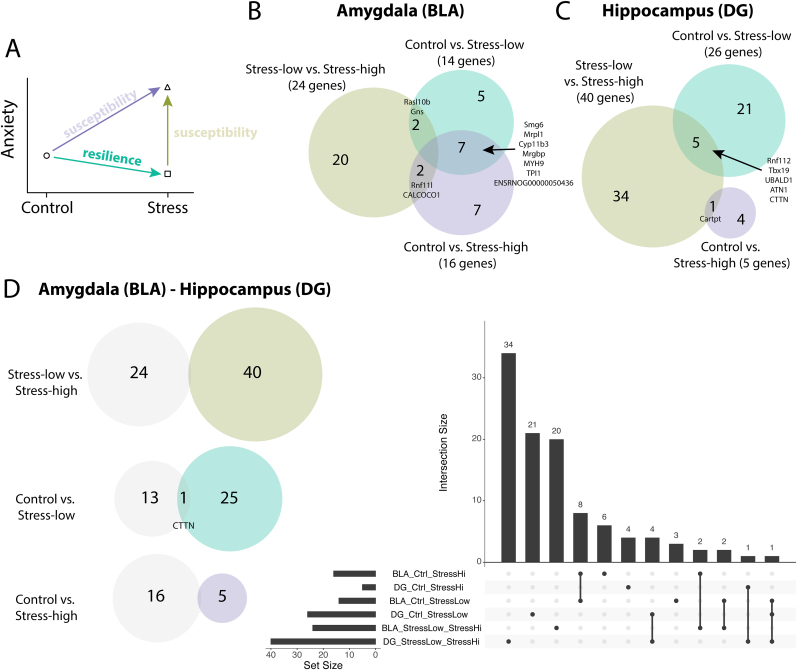
Regionally distinct transcriptomic changes in the basolateral amygdala and dentate gyrus following acute, severe stress. A. Schematic for differential gene expression (DGE) analysis. Arrows denote the pairwise comparisons conducted. B. Venn diagram showing DGE results for the basolateral amygdala (BLA) for our 3 pairwise comparisons. C. Venn diagram showing DGE analysis results for the hippocampal dentate gyrus (DG). D. Left, Venn diagram showing the overlap in differentially expressed genes between BLA and DG respective pairwise comparisons. Right, upset plot displaying overlaps between the 3 pairwise comparisons in the BLA versus DG.

We examined the molecular signatures in the amygdala and hippocampus that may contribute to divergent behavioral responses of rats to a single, severe stressor. PTSD is characterized by altered structure and function of limbic regions such as the amygdala and hippocampus (McEwen et al., 2016; Vyas et al., 2002; Mitra et al., 2005; Fa et al., 2014; Kirby et al., 2013), and both acute and chronic stressors induce transcriptional changes in these regions that correspond with resilient and vulnerable phenotypes in animal models (von Ziegler et al., 2022; Lori et al., 2019; Shen et al., 2019; Ponomarev et al., 2010; Datson et al., 2013; Sillivan et al., 2017; Daskalakis et al., 2014). We selected animals from either end of the phenotypic spectrum of anxiety-like behavior. Specifically, we selected control and stress-exposed rats with low and high composite anxiety-like scores (“stress-low” and “stress-high,” respectively) (Fig. 1, Fig. 2A). Of the selected animals, mean composite anxiety-like behavior scores were 4.4 ± 1.2 for control animals, 1.0 ± 0.58 for stress-low animals, and 14.0 ± 0.58 for stress-high animals. Stress-high composite scores were significantly greater than control scores (significant one-way ANOVA: F(2,8) = 31.79, p = 0.0002; Dunnet's multiple comparison of control versus stress-high, adjusted p = 0.0005), and we considered these animals as “vulnerable” to prolonged anxiety-like behavior after stress. In contrast, stress-low scores did not significantly differ from control scores (post hoc Dunnet's multiple comparison of control versus stress-low, adjusted p = 0.1026); thus, we considered these animals as “resilient.”

We conducted RNA-sequencing (RNA-seq) on bulk tissue isolated from the left hippocampal dentate gyrus (DG) and left basolateral amygdala (BLA) at the conclusion of behavior testing (Fig. 1A). This provides a snapshot of the delayed molecular correlates of divergent behavior outcomes after acute trauma. The left hemisphere was selected for standardization and consistency across animals. Principal component analysis (PCA) of normalized gene expression values revealed no clear clustering of control, stress-low, and stress-high animals (Fig. S1A–D), which suggests that our stressor induced moderate or subtle changes in gene expression in these regions. We thus carried out differential gene expression analysis to highlight group-specific differences. Pairwise comparisons between our three conditions yielded small sets of differentially expressed genes (DEGs; FDR <0.05) in both regions (Fig. 2B–C), suggesting that select genes may contribute to divergent behavioral outcomes.

We then asked whether the sets of DEGs were similar between the hippocampus and amygdala. We found little overlap between the sets of DEGs from the 10.13039/100011852BLA and the DG (Fig. 2D), supporting the idea that distinct mechanisms underlie stress-induced structural and functional changes in these two regions. Together, these results indicate that resilience and vulnerability to acute, severe stress are associated with modest, region-specific, long-term transcriptional differences in the BLA and DG.

### 3.3 Acute, severe stress affects expression of genes related to insulin and hormone signaling in the basolateral amygdala of susceptible and resilient animals

**Fig. 3:**
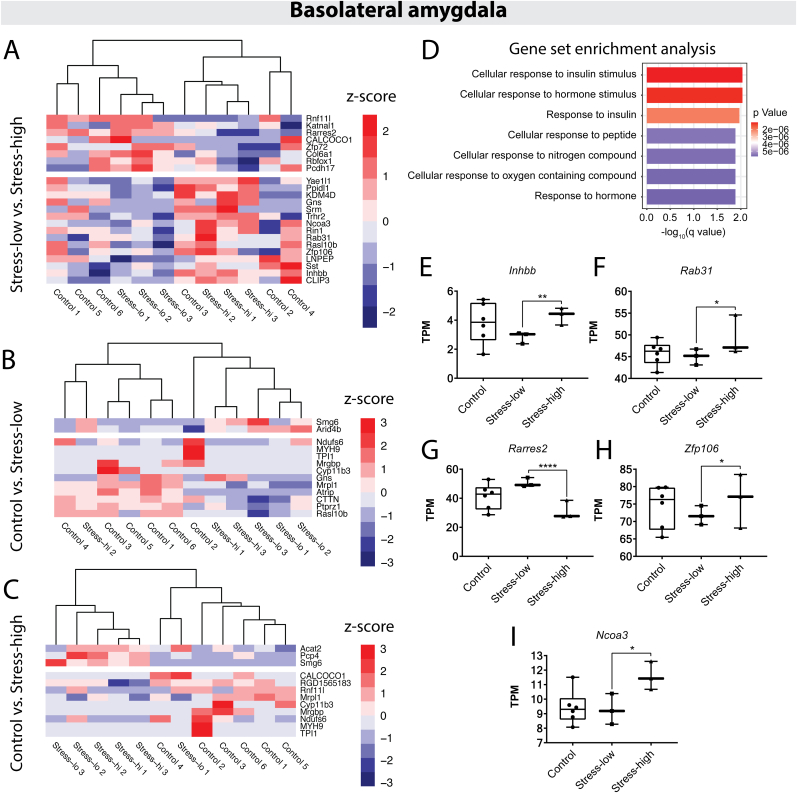
Gene expression changes in the basolateral amygdala of susceptible and resilient animals following acute, severe stress. A. Heatmap showing differentially expressed genes (DEGs) for the stress-low versus stress-high comparison. For all heatmaps: dendrograms show the clustering of samples, with sample group and number denoted at the bottom of the columns. Genes were clustered based on Pearson correlation, but dendrograms were omitted for clarity of presentation. Breaks denote the separation between up- and downregulated genes. Only genes with mapped gene names are shown. B. Heatmap of DEGs for the control versus stress-low comparison. C. Heatmap of DEGs for the control versus stress-high comparison. D. Results of Gene Set Enrichment Analysis (GSEA) showing the top significantly enriched Gene Ontology categories for the stress-low vs. stress-high comparison. E-I. Gene expression (in transcripts per million [TPM]) for genes comprising the top 2 Gene Ontology Biological Process categories. Asterisks denote significance after controlling for the false discovery rate (i.e., 1 - Posterior probability of being differentially expressed (PPDE)); ∗ ≤ 0.05, ∗∗ ≤ 0.01, ∗∗∗∗ ≤ 0.0001.

Given the separable transcriptional profiles of the amygdala and hippocampus in resilient and vulnerable animals, we sought to understand the individual genes and biological processes in these regions that might contribute to anxiety-like phenotypes. In the BLA, pairwise comparisons between the groups indicated that behavioral outcomes amongst stress-exposed animals yielded the largest separation in transcriptional profiles. Specifically, the comparison between stress-low and stress-high animals yielded 24 DEGs total (23 with assigned gene names, shown in Fig. 3A). Comparisons between control and either stress group returned few and largely overlapping DEGs (Fig. 3B–C). Hence, stress exposure in itself is less of a predictor of transcriptomic shift in the BLA compared to the long-term behavioral responses after stress.

Of the 23 DEGs with assigned names in the stress-low to stress-high comparison, 8 genes were downregulated and 15 upregulated in stress-high animals. To determine biological processes related to our observed anxiety-like phenotype, we conducted Gene Set Enrichment Analysis (GSEA)(Mootha et al., 2003; Subramanian et al., 2005) on these 23 DEGs. We found significant enrichment for gene ontology biological process (GOBP) categories including ‘cellular response to insulin stimulus’ and ‘cellular response to hormone stimulus’ (Fig. 3D). In addition to their role in response to insulin or hormone stimulus, 3 of the genes in the top two GOBP categories are specifically associated with modulation of anxiety-like behaviors in mice (*Inhbb* (Ageta et al., 2008; Zheng et al., 2009; Link et al., 2016), *Rab31* (Rouillard et al., 2016), and *Ncoa3* (Sun and Xu, 2020; Stashi et al., 2013; York et al., 2013)). All 3 genes showed significantly greater expression in stress-high animals compared to stress-low animals (Fig. 3E, F, I). Together, this may suggest that differences in amygdala insulin signaling, whether pre-existing or altered by stress exposure, correspond with differential resilience and vulnerability to anxiety-like behavior after stress.

### 3.4 Increased Cartpt expression in the dentate gyrus correlates with susceptibility after acute, severe stress exposure

**Fig. 4:**
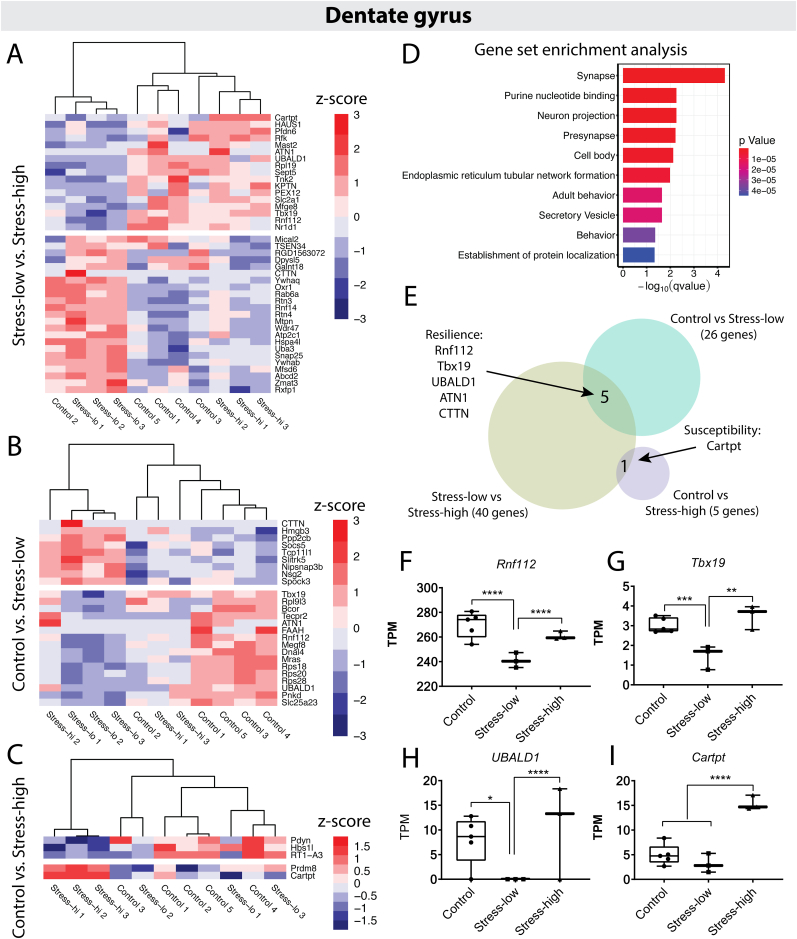
Gene expression changes in the hippocampal dentate gyrus of susceptible and resilient animals following acute, severe stress. A. Heatmap showing differentially expressed genes (DEGs) for the stress-low versus stress-high comparison. For all heatmaps: dendrograms show the clustering of samples, with sample group and number denoted at the bottom of the columns. Genes were clustered based on Pearson correlation, but dendrograms were omitted for clarity of presentation. Breaks denote the separation between up- and downregulated genes. Only genes with mapped gene names are shown. B. Heatmap of DEGs for the control versus stress-low comparison. C. Heatmap of DEGs for the control versus stress-high comparison. D. Results of Gene Set Enrichment Analysis (GSEA) showing the top significantly enriched Gene Ontology categories for the stress-low vs. stress-high comparison. E. Venn diagram displaying genes in the overlaps between the stress-low vs. stress-high and control vs. stress-low comparisons (‘Resilience’ signature genes) and stress-low vs. stress-high and control vs. stress-high comparisons (‘Susceptibility’ signature gene). F-H. Gene expression (in transcripts per million [TPM]) for *Rnf112*, *Tbx19* and *UBALD1*. I. *Cartpt* gene expression (in TPM). Asterisks denote significance after controlling for the false discovery rate (i.e., 1 - Posterior probability of being differentially expressed (PPDE)); ∗ ≤ 0.05, ∗∗ ≤ 0.01, ∗∗∗*p* ≤ 0.001, ∗∗∗∗ ≤ 0.0001.

The DG of the hippocampus is a brain region that is sensitive to stress, and glial composition of the DG correlates with anxiety-like behavior profiles following acute, severe stress exposure (Fa et al., 2014; Datson et al., 2013; Kirby et al., 2013; Long et al., 2021). We therefore examined whether differential gene expression in the DG of the hippocampus correlates with behavior outcomes after stress. Here, the comparison between stress-low and stress-high animals again yielded the largest separation in transcriptional profiles with 40 DEGs (Fig. 4A). The comparison between control and stress-low animals yielded 25 DEGs with assigned names (Fig. 4B), and the comparison between control and stress-high animals yielded only 5 DEGs (Fig. 4C). Thus, as observed in the BLA, the greatest differences in gene expression in the DG corresponded with differential long-term behavioral consequences of severe stress.

We next asked whether sets of genes contributing to specific biological processes are enriched in each of our group comparisons. GSEA revealed enrichment for biological categories related to neuronal structure and function, including ‘synapse’, ‘neuron projection’ and ‘presynapse’ (Fig. 4D). To gain a clearer understanding of the specific synaptic and functional processes involved, we present all individual DG DEGs and their currently known functions in Table 1, Table 2, Table 3. Many of the genes identified in the stress-low vs. stress-high comparison are associated with dendritic spine regulation, neurite outgrowth, and neuropsychiatric traits such as anxiety and mood. Thus, key genes implicated in neuronal plasticity and anxiety are differentially expressed between stress-low and stress-high animals.

**Table 1:** Differentially expressed genes between stress-low and stress-high animals in the dentate gyrus.

Gene Symbol	Gene Name	Adjusted *P*-value	Related Function	References
*RGD1563072*	Similar to hypothetical protein FLJ38984	0.0000	Unknown; high expression in brain	Entrez Gene: RGD1563072, similar to hypothetical protein FLJ38984
*CTTN*	Cortactin	0.0000	Actin cytoskeleton organization and cell shape; regulates activity-dependent synaptic plasticity at the Drosophila neuromuscular junction, neuronal spine actin dynamics and spatial memory formation in mice	Entrez Gene: CTTN, cortactin; Alicea et al., 2017; Cornelius et al., 2021
*Ywhaq*	Tyrosine 3-Monooxygenase/Tryptophan 5-Monooxygenase Activation Protein Theta (also 14-3-3 protein theta)	0.0000	Role in modulation of synaptic plasticity and neuronal development; YWHA-gene family proteins have been implicated in neuropsychiatric disorders including schizophrenia and bipolar disorder	Cornell and Toyo-oka, 2017; Berg et al., 2003; Foote and Zhou, 2012
*Rtn3*	Reticulon 3	0.0000	Neuronal Rtn family isoforms can inhibit neurite outgrowth; implicated in enhancing axonal regeneration; highly enriched in dystrophic neurites that surround amyloid B plaques in Alzheimer's disease brains	Shi et al., 2017; Alhajlah et al., 2021; Oertle and Schwab, 2003
*Cartpt*	Cocaine and Amphetamine Regulated Transcript Prepropeptide	0.0000	Well-established role in sensory processing, reinforcement/reward, anxiety, depression; missense mutation associated with increased anxiety in humans; hippocampal expression is modulated by stress	Hunter et al., 2007; Subhedar et al., 2014; Miraglia del Giudice, 2006; for review see Stanek, 2006
*UBALD1*	UBA Like Domain Containing 1	0.0000	Currently unknown	
*ATN1*	Atrophin 1	0.0000	Putative transcriptional co-repressor; found in nuclear, cytoplasmic compartments of neurons; intranuclear aggregates can lead to cell toxicity; mutations in ATN1 are associated with a rare neurodegenerative disorder (Dentatorubral-pallidoluysian atrophy (DRPLA))	Wood et al., 2000; Shen and Peterson, 2008
*Sept5*	Septin 5	0.0000	Deficiency in mice results in decreased anxiety-related behaviors and other deficits in affective/cognitive processes	Entrez gene: SEPTIN5, septin 5; Suzuki et al., 2009
*Mfge8*	Milk Fat Globule-EGF Factor-8	0.0000	One of most highly expressed, astrocyte-specific genes; neuroprotective role through modulation of inflammation, oxidative stress, and apoptosis in cerebral ischemia and neurodegenerative disease	Entrez Gene: Mfge8, milk fat globule EGF and factor V/VIII domain containing; Gao et al., 2018; Cheyuo et al., 2019; Cahoy et al., 2008
*TSEN34*	tRNA-Splicing Endonuclease Subunit 34	0.0000	Mutation in this gene is associated with the neurological disorder pontocerebellar hypoplasia type 2	Entrez gene: Tsen34, tRNA splicing endonuclease subunit 34; Hayne et al., 2020; Namavar et al., 2011
*Rnf112*	Ring Finger Protein 112 (also Zinc Finger Protein 179 (ZNF179))	0.0000	Primarily expressed in the brain; involved in regulation of dendritic spine density, synaptic transmission	Lomash et al., 2015; Matsuda et al., 1996
*Mical2*	Microtubule Associated Monooxygenase, Calponin And LIM Domain Containing 2	0.0000	MICALs link cytoskeleton dynamics, vesicle trafficking, synaptic structure and redox signaling; role in regulation of synaptic and mitochondrial pathways underlying stress, sleep and neuropathology	Zhou et al., 2011; Jiang et al., 2015
*Mfsd6*	Major Facilitator Superfamily Domain Containing 6	0.0000	Abundantly expressed in mouse brain neurons but not astrocytes; putative role in regulating neural circuit activity	Bagchi et al., 2020; McCulloch et al., 2017
*Rnf14*	E3 Ubiquitin-protein Ligase Ring Finger Protein 14	0.0001	Recently described as a novel tau E3 ligase involved in degradation of pathological tau in an Alzheimer's disease (AD) mouse model (ADLPAPT) and AD patient-derived organoids	Entrez Gene: Rnf14, ring finger protein 14; Choi et al., 2020
*PEX12*	Peroxisomal Biogenesis Factor 12	0.0001	Mutations are associated with peroxisome biogenesis disorders (PBDs) - a group of autosomal recessive developmental brain disorders	Gootjes et al., 2004); Steinberg et al., 2006; Steinberg et al., 1993; Steinberg et al., 2004
*Tnk2*	Tyrosine Kinase Non Receptor 2 (also Activated Cdc42-associated kinase 1 (ACK1))	0.0001	High expression in brain with important role in neurotrophin signaling, neuritic outgrowth and arborization; mutations associated with infantile onset epilepsy and intellectual disability	Entrez Gene: TNK2, tyrosine kinase non receptor 2; Urena et al., 2005; La Torre et al., 2006; La Torre et al., 2013; Hitomi et al., 2013
*Hspa4l*	Heat Shock Protein Family A (Hsp70) Member 4 Like	0.0003	Function in brain currently unknown; in silico study suggests upregulation in several brain regions (including hippocampus) of patients with Multiple sclerosis, associated with myelination and immune system function	Chiricosta et al., 2020
*Nr1d1*	Nuclear Receptor Subfamily 1 Group D Member 1	0.0003	Linked to regulation of mood-related behaviors and pathologies related to social deficits; knockdown of Nr1d1 in the Nucleus accumbens of female mice reduced anxiety and increased sociability (no significant behavioral effects observed in males)	Entrez Gene: Nr1d1, nuclear receptor subfamily 1, group D, member 1; Zhao and Gammie, 2018
*Rpl19*	Ribosomal Protein L19	0.0007	Encodes a ribosomal protein that is a component of the 60S (large) subunit; upregulated in hypothalamus (but not hippocampus) of male mice subjected to chronic social defeat stress	Smagin et al., 2015
*Rxfp1*	Relaxin Family Peptide Receptor 1	0.0007	Role in stress responses, memory and emotional processing	Tanaka, 2010; Lee et al., 2016
*Snap25*	Synaptosomal-Associated Protein, 25 kDa	0.0017	Component of the SNARE complex which is essential for synaptic vesicle exocytosis; involved in synaptogenesis, neurotransmitter release, spine morphogenesis, post-synaptic vesicle trafficking, neural plasticity; deficiency or alterations in SNAP25 function have been shown to induce anxiety in mice	Kataoka et al., 2011; Irfan et al., 2019
*Mtpn*	Myotrophin	0.0029	Potential function in cerebellar neuron differentiation and cerebellar morphogenesis	Entrez Gene: MTPN, myotrophin
*Abcd2*	ATP-binding Cassette Sub-family D Member 2	0.0051	Mutations in this gene have been observed in patients with adrenoleukodystrophy, a severe demyelinating disease	Entrez Gene: ABCD2, ATP binding cassette subfamily D member 2
*Rfk*	Riboflavin Kinase	0.0052	Human Rfk has been recently linked to neurological diseases and regulation of circadian rhythms	Karthikeyan et al., 1993; Kukharsky et al., 2020; Johnson et al., 2012; Hirano et al., 2017
*Galnt18*	Polypeptide N-Acetylgalactosaminyltransferase 18	0.0080	Role in mucin type O-glycan biosynthesis and protein metabolism; CNS-specific role unknown	Entrez Gene: GALNT18, polypeptide N-acetylgalactosaminyltransferase 18; Shan et al., 2019
*Tbx19*	T-box Transcription Factor 19	0.0083	Involved in the corticotropin-releasing hormone (CRH) signaling pathway - a central part of the hypothalamic-pituitary-adrenal (HPA) or ‘hormonal’ stress axis	Liu et al., 2001
*Atp2c1*	ATPase Secretory Pathway Ca2+ Transporting 1	0.0108	Role in Ca(2+) homeostasis in trans-Golgi compartment which impacts Golgi and trans-Golgi protein sorting	Entrez Gene: ATP2C1, ATPase secretory pathway Ca2+ transporting 1; Lissandron et al., 2010
*Ywhab*	Tyrosine 3-Monooxygenase/Tryptophan 5-Monooxygenase Activation Protein Beta (also 14-3-3 protein beta)	0.0112	YWHA-gene family proteins have been implicated in neuropsychiatric disorders including schizophrenia	Cornell and Toyo-oka, 2017; Berg et al., 2003; Foote and Zhou, 2012
*Rab6a*	RAB6A, member RAS oncogene family	0.0123	Canonical role in targeting and fusion of transport carriers to acceptor compartments; differentially expressed in the basolateral amygdala complex (BLC) of mice following acute restraint stress	Entrez Gene: Rab6a, RAB6A, member RAS oncogene family; Sillivan et al., 2019
*KPTN*	Kaptin, Actin binding protein	0.0150	Important role in neuromorphogenesis; mutations in this gene result in an autosomal recessive form of intellectual disability; diseases associated to KPTN also include familial, temporal lobe epilepsy	Entrez Gene: KPTN, kaptin, actin binding protein; Thiffault et al., 2020; Pajusalu et al., 2015
*Wdr47*	WD Repeat Domain 47	0.0151	Controls neuronal polarity by regulating local microtubule dynamics; important role in brain development and autophagy	Chen et al., 2020; Kannan et al., 2017
*Rtn4*	Reticulon 4 (also Neurite Outgrowth Inhibitor (Nogo))	0.0181	Isoform A inhibits neurite outgrowth, branching and extension during development; additionally regulates radial migration of cortical neurons and is a negative regulator of nervous system angiogenesis; isoform B: mainly functions in endothelial cells and regulates vascular remodeling	Entrez Gene: RTN4, reticulon 4; GrandPré et al., 2000; Petrinovic et al., 2010(); Wälchli et al., 2013; Acevedo et al., 2004; Jia et al., 2016
*Oxr1*	Oxidation Resistance Protein 1	0.0186	Shown to protect neurons from oxidative stress damage in animal models of amyotrophic lateral sclerosis (ALS) and neurodegeneration	Entrez Gene: Oxr1, oxidation resistance 1; Liu et al., 2015; Volkert and Crowley, 2020
*Dpysl5*	Dihydropyrimidinase Like 5 (also Collapsin Response Mediator Protein 5 (CRMP5))	0.0226	Role in neuronal migration, axonal guidance, dendrite outgrowth, and synapse formation through its interaction with microtubules; modulates susceptibility to chronic social defeat stress in mice	Entrez Gene: DPYSL5, dihydropyrimidinase like 5; Jeanne et al., 2021; Quach et al., 2015; Lin et al., 2021
*Uba3*	Ubiquitin Like Modifier Activating Enzyme 3	0.0248	Involved in ubiquitin-like NEDD8 conjugation pathway (neddylation); CNS-specific function unknown	Entrez Gene: Uba3, ubiquitin like modifier activating enzyme 3
*HAUS1*	HAUS Augmin Like Complex Subunit 1	0.0333	Necessary for mitotic spindle assembly; CNS-specific function unknown	Entrez Gene: HAUS1, HAUS augmin like complex subunit 1
*Mast2*	Microtubule Associated Serine/Threonine Kinase 2	0.0359	Diseases associated with Mast2 include Mega-Corpus-Callosum Syndrome with Cerebellar Hypoplasia And Cortical Malformations	Rebhan et al., 1997, https://www.genecards.org/cgi-bin/carddisp.pl?gene=MAST2
*Pfdn6*	Prefoldin Subunit 6	0.0394	Component of molecular chaperone prefoldin-like complex; related gene ontology (GO) pathways include unfolded protein binding, chaperone binding	Entrez Gene: PFDN6, prefoldin subunit 6; Son et al., 2018
*Slc2a1*	Solute Carrier Family 2 Member 1 (also Glucose transporter 1, GLUT1)	0.0404	Most important energy carrier in the mammalian brain; sexually-dimorphic expression observed in hippocampus of adolescent rats following chronic stress exposure	Entrez Gene: SLC2A1, solute carrier family 2 member 1; Kelly et al., 2014
*Zmat3*	Zinc Finger Matrin-Type 3 (also Wild Type p53-induced Gene 1(Wig-1))	0.0411	Key splicing regulator of p53 tumor suppression pathway; high expression in the brain (amygdala, prefrontal cortex); rat ortholog (PAG608) shows constitutively high expression in various regions of the rat nervous system and is induced by a number of stress agents (ischemia, treatment with methamphetamine, ALS disease onset, L-DOPA in a Parkinson's disease model)	Bieging-Rolett et al., 2020); Vilborg et al., 2011; (Gillardon et al., 1999; Asanuma et al., 2007; Morimoto et al., 2010; Shimizu et al., 2008

**Table 2:** Differentially expressed genes between stress-low and control animals in the dentate gyrus.

Gene Symbol	Gene Name	Adjusted *P*-value	Related Function	References
*CTTN*	Cortactin	0.0000	Role in actin cytoskeleton organization, cell shape; regulates activity-dependent synaptic plasticity (Drosophila NMJ) and neuronal spine actin dynamics, spatial memory formation in mice	Entrez Gene: CTTN, cortactin; Alicea et al., 2017; Cornelius et al., 2021
*Mras*	Muscle RAS Oncogene Homolog	0.0000	Member of RAS family of small GTPases, role in signal transduction mediating cell growth and differentiation; knockout of M-Ras in mice resulted in deficits in social interaction, increased fear-related behavior	Entrez Gene: MRAS, muscle RAS oncogene; Ehrhardt et al., 2015
*Megf8*	Multiple EGF Like Domains 8	0.0000	Role in Bone Morphogenetic Protein 4 (BMP4) signaling in trigeminal ganglion neurons, important for axon guidance in the peripheral nervous system	Engelhard et al., 2013
*ATN1*	Atrophin 1	0.0000	Putative role as transcriptional co-repressor; expressed in neurons; intranuclear aggregates can lead to cell toxicity; ATN1 mutations are associated with a rare neurodegenerative disorder (Dentatorubral-pallidoluysian atrophy (DRPLA))	Wood et al., 2000; Shen and Peterson, 2008
*Rnf112*	Ring Finger Protein 112 (also Zinc Finger Protein 179 (ZNF179))	0.0000	Member of the RING finger protein family of transcription factors; primary expression in brain; involved in regulation of dendritic spine density, synaptic transmission	Lomash et al., 2015; Matsuda et al., 1996
*FAAH*	Fatty Acid Amide Hydrolase	0.0000	Encodes protein involved in primary and secondary fatty acid amide hydrolysis (including neuromodulatory anandamide, oleamide); modulation of anandamide signaling (via FAAH) is a therapeutic target for stress-related neuropsychiatric diseases	Entrez Gene: FAAH, fatty acid amide hydrolase; Gunduz-Cinar et al., 2013; Morena et al., 2019
*Spock3*	SPARC/Osteonectin, Cwcv and Kazal-Like Domains Proteoglycan 3	0.0000	Related pathways include extracellular matrix degradation and ERK signaling; putative risk gene for adult ADHD and personality disorders	Entrez Gene: SPOCK3, SPARC (osteonectin), cwcv and kazal like domains proteoglycan 3; Weber et al., 2014
*Rpl19*	Ribosomal protein L9-like	0.0001	Predicted component of the 60S large ribosomal subunit; involved in cellular response to nerve growth factor (NGF) stimulus; upregulated in response to NGF in PC12 cells, suggesting it plays a role in neuronal differentiation	Angelastro et al., 2002
*Pnkd*	Paroxysmal Nonkinesiogenic Dyskinesia (PNKD) Metallo-Beta-Lactamase Domain Containing	0.0002	Mutations are associated to the movement disorder paroxysmal non-kinesigenic dyskinesia (characterized by involuntary unilateral or bilateral movements); association with Tourette Disorder or Tic disorder	Erro, 1993; Sun et al., 2018
*Tcp11l1*	T-Complex 11 Like 1	0.0002	Currently unknown	Entrez Gene: TCP11L1, t-complex 11 like 1
*Tbx19*	T-box Transcription Factor 19	0.0007	Involved in corticotropin-releasing hormone (CRH) signaling pathway - a central part of the hypothalamic-pituitary-adrenal (HPA) or ‘hormonal’ stress axis	Liu et al., 2001
*Bcor*	BCL6 Corepressor	0.0017	Interacting partner for the BCL6 oncogenic repressor; associated with neuroepithelial tumors	Entrez Gene: BCOR, BCL6 corepressor; Bunting and Melnick, 2013; De Lima et al., 2020
*Slitrk5*	SLIT and NTRK Like Family Member 5	0.0021	Member of Slitrk family of proteins, involved in synaptogenesis, neurite outgrowth, neuronal survival; modulates Brain-Derived Neurotrophic Factor (BDNF)-dependent pathways via interaction with TrkB receptors; modulates hyperactivity behavior	Yim et al., 2013); Song et al., 2015 ; Shmelkov et al., 2010; Salesse et al., 2020
*Rps28*	Ribosomal Protein S28	0.0022	Component of the 40S (small) ribosomal subunit; likely involved in ribosome biogenesis	Kim et al., 2017
*Socs5*	Suppressor Of Cytokine Signaling 5	0.0028	Member of SOCS (also known as STAT-induced STAT inhibitor (SSI)) family of proteins; associated with cell proliferation and tumorigenesis	Entrez Gene: SOCS5, suppressor of cytokine signaling 5; Trengove and Ward, 2013; Sharma et al., 2019; Sanchez-Mejias et al., 2019
*Rps18*	Ribosomal Protein S18	0.0039	Component of the 40S (small) ribosomal subunit; involved in initiation of translation	Entrez Gene: RPS18, ribosomal protein S18
*Slc25a23*	Solute Carrier Family 25 Member 23	0.0052	Calcium-dependent mitochondrial solute carrier	Bassi et al., 2005
*Dnal4*	Dynein Axonemal Light Chain 4	0.0061	Functions in outer dynein arms complex - a molecular motor that produces force to move cilia; associated with Congenital mirror movement disorder	Entrez Gene: DNAL4 dynein, axonemal, light chain 4; Ahmed et al., 2014
*UBALD1*	UBA Like Domain Containing 1	0.0111	Currently unknown	
*Hmgb3*	High Mobility Group Box 3	0.0120	Member of protein family with one or more high mobility group DNA-binding motifs; important role in maintaining stem cell populations	Entrez Gene: HMGB3, high mobility group box 3; Nemeth et al., 2006
*Rps20*	Ribosomal Protein S20	0.0169	Component of the 40S (small) ribosomal subunit; role in cell proliferation and tumor development	Krishnan et al., 2018; Goudarzi, 2016
*Tecpr2*	Tectonin Beta-Propeller Repeat-Containing 2	0.0213	Role in autophagy-related neurodegenerative disorder spastic paraplegia type 49 (SPG49), characterized by intellectual disability, decreased pain sensitivity, autonomic-sensory neuropathy, chronic respiratory disease	Oz-Levi et al., 2012; Tamim-Yecheskel et al., 2020
*Unknown*	NA	0.0223	NA	NA
*Nipsnap3b*	Nipsnap Homolog 3B	0.0280	Member of protein family with possible role in vesicular trafficking	Buechler et al., 2004
*Nsg2*	Neuronal Vesicle Trafficking Associated 2	0.0390	Neuron-specific protein involved in modulation of excitatory neurotransmission	Chander et al., 2019

**Table 3:** Differentially expressed genes between stress-high and control animals in the dentate gyrus.

Gene Symbol	Gene Name	Adjusted *P*-value	Related Function	References
*Cartpt*	Cocaine and Amphetamine Regulated Transcript Prepropeptide	0.0000	Role in sensory processing, reinforcement, reward, anxiety, depression; missense mutation associated with increased anxiety in humans; hippocampal expression is modulated by stress	Hunter et al., 2007; Subhedar et al., 2014; Miraglia del Giudice, 2006; for review see Stanek, 2006
*Prdm8*	PR/SET Domain 8	0.0000	Member of conserved family of histone methyltransferases that negatively regulate transcription; involved in neural circuit assembly and neocortical development	Ross et al., 2012; Inoue et al., 2014
*RT1-A3*	RT1 class I, locus A3 (also MHC class I protein)	0.0003	Role in immune system antigen presentation; expression modulated in hippocampus after chronic immobilization stress	VanGuilder Starkey et al., 2012; Li et al., 2013
*Hbs1l*	Hsp70 Subfamily B Suppressor 1-Like Protein	0.0101	Member of GTP-binding elongation factor family; associated with regulation of fetal hemoglobin; no known CNS-specific function	Entrez Gene: HBS1L, HBS1 like translational GTPase; Stadhouders et al., 2014
*Pdyn*	Prodynorphin	0.0403	Encodes a preprotein that yields several active opium-like peptides which are ligands for the kappa-type opioid receptor; ligand functions are associated to stress, anxiety, depression, pain	Entrez Gene: PDYN, prodynorphin; Baird et al., 2021; Bodnar, 2013 ; Szklarczyk et al., 2012; Johnson et al., 2021

To obtain a common set of candidate genes that may drive resilience versus susceptibility, we examined the genetic overlap between each of the 3 pairwise comparisons (Fig. 4E). We identified genes common to resilience by comparing the gene sets from the control vs. stress-low and stress-low vs. stress-high comparisons. We found 5 overlapping genes: *Rnf112* (*Ring finger protein 112; Zinc finger protein 179* [*ZNF179*]; *Neurolastin*), *Tbx19* (*T-box transcription factor 19*), *UBALD1* (*UBA Like Domain Containing 1*), *ATN1* (*Atrophin 1*), and *CTTN* (*Cortactin*). Differential expression of *ATN1* and *CTTN* was driven by outlier values (Fig. S5); thus, we disregarded these genes. Notably, individual expression values for *Rnf112* (Fig. 4F), *Tbx19* (Fig. 4G), and *UBALD1* (Fig. 4H) revealed that expression of each of these genes was significantly downregulated in stress-low animals when compared to both control and stress-high animals. Additionally, hierarchical clustering by animal from the stress-low vs. stress-high comparison (Fig. 4A) indicated that stress-high animals clustered with the majority of control animals. This indicates that stress-high animals display gene expression profiles that are more similar to controls than stress-low animals. These findings may suggest that an active transcriptional response promotes resilience to acute, severe stress.

We next identified common susceptibility genes by examining the overlap between DEGs from the control vs. stress-high and stress-low vs. stress-high comparisons. This revealed a single shared gene — *Cartpt* (cocaine and amphetamine regulated transcript [CART] prepropeptide). Transcription of *Cartpt* was significantly upregulated in the stress-high group compared to both control and stress-low animals (Fig. 4I), while stress-low values did not differ from controls. This indicates that *Cartpt* expression in the DG may serve as a marker or driver of enhanced anxiety-like behavior after acute, severe stress. We therefore asked whether *Cartpt* expression levels relate to anxiety-like behavior scores amongst all individual animals. Interestingly, *Cartpt* expression was positively correlated with composite anxiety-like behavior scores and avoidance sub-scores, but not startle sub-scores (Fig. S6). Future work should seek to validate expression of *Cartpt* via real-time PCR and *in situ* hybridization to confirm upregulation of this gene in stress-high animals and to determine its cell type-specific pattern of expression in the DG.

Taken together, these analyses indicate that a distinct set of genes is altered in the hippocampal DG following acute, severe stress. Furthermore, as with the BLA, the largest number of differentially expressed genes was associated with differential long-term behavioral resilience or susceptibility to anxiety-like behavior after stress. Interestingly, patterns of expression of key genes strongly corresponded with stress resilience and vulnerability and may point to novel mechanisms underlying stress-induced plasticity that prevent or promote anxiety-like behavior.

## 4 Discussion

In this study, we identified transcriptional signatures in the hippocampus and amygdala that correspond with different patterns of anxiety-like behavior after a single, severe stressor. One week after acute stress, rats displayed substantial variability in avoidance and startle. A subset of animals exhibited high anxiety-like behavior that correlated with sustained corticosterone release during the stress event. Bulk RNA sequencing revealed that distinct sets of genes in the amygdala and hippocampus were associated with behavioral outcomes after stress, suggesting that separable mechanisms underlie stress-induced functional changes in these two regions. In the basolateral amygdala (BLA), gene sets related to insulin and hormone signaling were largely upregulated in vulnerable animals compared to resilient animals. In the hippocampal dentate gyrus (DG), gene set enrichment analysis highlighted categories related to synaptic structure and neuronal projections. Stress vulnerability in the DG strongly corresponded with upregulation of *Cartpt*, a corticosterone-regulated gene, while stress resilience corresponded with downregulation of *Rnf112* and *Tbx19*. Altogether, these findings provide a novel perspective on hippocampal and amygdala molecular regulation of divergent behavioral outcomes after trauma.

Prior studies suggest that endocrine and physiological responses to stress can predict future behavioral outcomes (Cohen et al., 2006; McFarlane et al., 1997; Shalev et al., 1998; Yehuda et al., 1998a; Monari et al., 2024; Hodes et al., 2014). Here, we found that corticosterone at the end, but not the beginning, of the 3-h stressor positively correlated with anxiety-like behavior scores one week later. This may suggest that sustained release of corticosterone during trauma contributes to long-lasting anxiety-like behavior (Chen et al., 2008; McEwen, 2005; Chetty et al., 2014). This finding contrasts with several studies suggesting that blunted corticosterone responses are correlated with PTSD or anxiety-like behavior outcomes (Cohen et al., 2006; Monari et al., 2024). Such discrepancies may arise from experimental differences in the rat strain examined, the type of stressor utilized, and the behavioral outcomes measured. Notably, corticosterone accounted for only 30 % of the variance of behavior scores. The causal relationship between corticosterone and subsequent behavior remains unknown, underscoring the need for further characterization of the association between immediate endocrine responses to stress and subsequent behavioral outcomes. Corticosterone may not act as a mechanistic determinant of long-term behavioral change, but rather as one contributing marker among many, and its utility in stratifying behavioral phenotypes remains limited based on our data. Analyzing corticosterone levels both before stress and at the time of behavioral profiling would add valuable insight into the dynamic relationship between glucocorticoid regulation and the long-term effects of stress on anxiety-like behavior.

We identified transcriptional signatures in key limbic subregions linked to behavioral outcomes after stress. Global gene expression of both the DG and BLA did not correlate with persistent anxiety-like behavior after acute stress. Rather, select gene sets distinguished control, resilient, and vulnerable animals. In the BLA, a small number of genes related to insulin or hormone responses separated vulnerable and resilient animals. Specifically, 3 of the genes (*Inhbb, Rab31, Ncoa3)* were upregulated in vulnerable animals compared to resilient animals. Interestingly, insulin and Insulin-like Growth Factor (IGF-1) signaling in the amygdala has been shown to affect synaptic function and to influence anxiety-like behavior in mice (Santi et al., 2018; Soto et al., 2019). Similarly, associations between insulin resistance, IGF-1, and mood disorders (i.e., anxiety) have been reported in humans (Bot et al., 2016; Santi et al., 2018 ). Hence, altered insulin signaling in the amygdala prior to or following acute stress exposure may predispose an individual to persistent anxiety-like behavior. Future studies should explore the role of amygdala-specific insulin signaling in both stress responses and anxiety.

Transcriptional profiles of the hippocampal DG differed from those of the BLA in resilient and vulnerable animals, consistent with prior studies and suggesting that region-specific molecular mechanisms regulate prolonged anxiety-like behavior (McEwen et al., 2016; Regev-Tsur et al., 2020). Few genes in the DG distinguished control animals from vulnerable animals. Rather, greater numbers of genes separated resilient animals from both control and vulnerable animals. These genes were enriched for synaptic plasticity biological processes, and many of them regulate dendritic spine structure and function (*Rnf112* (Lomash et al., 2015)*, Snap25* (Irfan et al., 2019)) as well as neurite outgrowth and arborization (*Tnk2* (La Torre et al., 2013)*, Dpysl5* (Quach et al., 2015)*, Rtn3, Rtn4* (Oertle and Schwab, 2003)). Dendritic remodeling in brain regions responsive to stress is a crucial mechanism underlying stress-induced changes in synaptic plasticity, connectivity, and behavior (McEwen, 1999; McEwen et al., 2016; Vyas et al., 2002; Leuner and Shors, 2013). We also identified differentially expressed genes involved in anxiety, mood, emotion (*Cartpt* (Stanek, 2006)*, Snap25* (Kataoka et al., 2011)*, Rxfp1* (Tanaka, 2010)*, Nr1d1* (Zhao and Gammie, 2018)*, Sept5* (Suzuki et al., 2009)), and neuropsychiatric disorders (YWHA-family (Foote and Zhou, 2012)). Expression patterns of many of these genes were altered specifically in resilient animals. This suggests that resilience involves active regulation of plasticity and mood-related pathways, adding to a growing body of work suggesting that resilience is not a lack of response, but an active means of protection from the effects of stress (Nasca et al., 2019; Richter-Levin et al., 2019; Krishnan et al., 2007; Russo et al., 2012).

Our approach of comparing control, resilient, and vulnerable animals allowed us to examine the overlap among pairwise comparisons to identify common resilience or vulnerability signatures in the DG. The resilience signature indicated that *Rnf112* and *Tbx19* are significantly downregulated in resilient animals. *Rnf112* enhances excitatory synaptic transmission in mice (Lomash et al., 2015)*,* while *Tbx19* is involved in the corticotropin-releasing hormone (CRH) signaling pathway — a central part of the hypothalamic-pituitary-adrenal (HPA) axis (Chen et al., 2012). Downregulation of these key genes in the hippocampus may thus serve to dampen excitation or stress axis reactivity to regulate anxiety-like behavior following a stressor, again suggesting that specific molecular mechanisms may be activated to protect an individual from long-term changes to mood and anxiety. The vulnerability signature consisted of a single gene, *Cartpt,* expression of which positively correlated with anxiety-like behavior. *Cartpt* encodes a multifaceted neuropeptide that plays a role in reward, sensory processing, anxiety, and depression in both humans and rodents (Stanek, 2006; Hunter et al., 2007; Subhedar et al., 2014; Miraglia del Giudice, 2006). Upregulation of *Cartpt* in the DG might thus serve as an indicator or driver of susceptibility to anxiety after an acute, severe stressor. Targeting these genes and validating differential expression in future experiments could pave the way to identifying novel therapeutic targets for the prevention of stress-induced mood and anxiety disorders.

Recent studies examining molecular changes across species, stress models, and longitudinal time points reveal both shared and distinct transcriptional responses to stress when compared to our findings. For example, chronic restraint stress in adolescent rats induces region- and sex-specific transcriptomic changes across nine brain regions, including downregulation of *Cartpt* in the arcuate nucleus of the hypothalamus in both sexes — contrasting our findings from the DG of vulnerable males — as well as *Sst* upregulation in CA1 of males — mirroring our findings from the BLA in vulnerable males (Krolick et al., 2024). Using a chronic unpredictable mild stress paradigm in adult rats, Yang et al. (2023) identified key genes associated with depression-like behaviors in the hippocampus and amygdala (Yang et al., 2023). Candidate genes in the amygdala enriched for myelination and oligodendrocyte function, consistent with our prior protein-level findings (Long et al., 2021); however, myelin genes were not enriched in the present study. In the hippocampus, one key candidate gene, *Mmp9*, was associated with synaptic plasticity, and while this specific gene was not differentially expressed in our study, synaptic plasticity was a common theme among hippocampal DEGs in resilient animals. Acute stress studies similarly highlight rapid shifts in gene expression tied to synaptic activity and transcriptional regulation. Within 45 min of an acute stressor, the hippocampus displays upregulation of genes related to transcriptional regulation, and downregulation of genes related to cell adhesion and cell junction (von Ziegler et al., 2022; Floriou-Servou et al., 2018). Fifteen minutes after cued fear conditioning in male mice, genes that enrich for synaptic activity are differentially expressed in the amygdala (Reis et al., 2022). Compared to these genes, 5 of our hippocampal genes (*Abcd2, Hmgb3, Pdyn, Prdm8, Spock3*) and 3 amygdala genes (*Pcdh17, Rasl10b, Rin1*) overlapped. Zhao et al. (2024) used SLAM-seq to detect nascent transcripts in the amygdala of male rats following a 2-h stressor and identified gene sets linked to synaptic function (Zhao et al., 2024), which are consistent with our findings. Additionally, microarray analysis of the hippocampus and amygdala from susceptible and resilient male rats revealed altered transcription related to glucocorticoid receptor signaling one week after predator scent stress, supporting the role of endocrine regulation in the long-term effects of stress (Daskalakis et al., 2014). Although the behavioral paradigm closely resembled that of this study, only one gene — *Pdyn* — was differentially expressed in the hippocampus of both studies. Interestingly, this gene, encoding prodynorphin, modulates anxiety and is regulated by both adrenal glucocorticoids and stress (Johnson et al., 2021; Thai et al., 1992; Wittmann et al., 2009). Together, these studies and our own highlight that stress-induced transcriptional programs are diverse and may depend on experimental factors such as species, developmental stage, type of stressor, tissue collection time point, and tissue identity (e.g., whole regions versus sub-nuclei). Despite these variables, there is some convergence at the level of biological processes — particularly synaptic function and plasticity.

The longitudinal expression patterns of the genes identified in this study remain unclear, as behavioral profiling after stress is necessary to classify resilient and vulnerable animals. Expression differences may arise before, during, or well after stress exposure and behavioral assessment. Novel imaging methods that allow for longitudinal, *in vivo* characterization of native transcription may be a valuable tool to map expression changes over time, and a key future direction of this work will be to conduct gain- or loss-of-function experiments at different time points to clarify causal roles for each gene. Despite these limitations, our findings provide a valuable foundation for generating hypotheses and exploring gene regulation underlying long-term stress responses.

Some limitations of our study include limited animal sequencing, sex specificity, and the need for gene validation. RNA sequencing was conducted on just three animals from each behavioral extreme to maximize behavioral contrasts. Expanding the sample size and including animals across the behavioral spectrum could increase statistical power and uncover more nuanced transcriptional patterns. Furthermore, we selected stress-exposed animals from the lower range of the distribution to represent “resilient” animals. Doing so may have selected for animals that simply do not show a strong response to the stressor, and choosing animals near the mean of the group may better represent a “resilient” phenotype. Second, our results are specific to males. We have noted that, in our hands, female Sprague Dawley rats do not respond to this combinatorial stressor with a spectrum of avoidance and startle behavior, making it impossible to identify resilient and vulnerable populations via these behavioral metrics. Given the prominent sex differences in PTSD diagnoses for human populations (Breslau, 2002; Breslau and Anthony, 2007; Christiansen and Berke, 2020; Pooley et al., 2018; Shansky, 2015), future experiments should expand this work to include females by identifying appropriate stressors and/or behavioral assays for female rats. Furthermore, validation of the genes we identified in this study will be crucial for future work in this domain. In this study, RNA yields were small and were exhausted in the sequencing process; hence, caution should be taken when interpreting the results of specific genes. Immunofluorescence will also be key to both validation and understanding of the cellular localization of gene expression. In addition, the goal of our stress paradigm was to identify animals with stress vulnerability to model PTSD; while anxiety is a core symptom cluster of PTSD, our results cannot distinguish whether our stress-vulnerable animals demonstrate PTSD-like behavior or generalized anxiety-like behavior. Lastly, stress-exposed animals were housed separately for two days after the stressor to prevent predator odor contamination in our colony room. Despite identical lighting, humidity, etc., this may have introduced mild environmental stress, though this was deemed preferable to permanently housing the control and stress groups in separate rooms.

In this work, we demonstrate that divergent behavioral outcomes after a single, severe stressor are associated with regionally distinct transcriptional profiles in the amygdala and the hippocampus. Key genes in the amygdala that are associated with insulin and hormonal signaling correspond with resilience and vulnerability. In the hippocampus, *Cartpt* was strongly correlated with vulnerability to anxiety-like behavior; however, transcriptional changes were primarily associated with stress resilience, suggesting that an active molecular response in the hippocampus facilitates protection from the long-term consequences of severe stress. These results provide novel insight into the molecular mechanisms that bring about individual variability in the behavioral responses to stress and provide new targets for the advancement of therapies for stress-induced neuropsychiatric disorders.

## CRediT authorship contribution statement

**Kimberly L.P. Long:** Writing – review & editing, Writing – original draft, Visualization, Validation, Software, Project administration, Methodology, Investigation, Formal analysis, Data curation, Conceptualization. **Sandra E. Muroy:** Writing – review & editing, Writing – original draft, Visualization, Validation, Software, Project administration, Formal analysis, Data curation. **Siamak K. Sorooshyari:** Writing – review & editing, Software, Formal analysis. **Mee Jung Ko:** Writing – review & editing, Investigation, Formal analysis. **Yanabah Jaques:** Writing – review & editing, Investigation. **Kishant Mohan:** Formal analysis. **Peter Sudmant:** Writing – review & editing, Supervision, Software, Resources, Methodology, Formal analysis, Conceptualization. **Daniela Kaufer:** Writing – review & editing, Supervision, Resources, Project administration, Funding acquisition, Conceptualization.

## Funding

This research was supported by the 10.13039/100000025National Institute of Mental Health [R01MH115020 to D.K.], the Berkeley Neuro-AI Resilience Research Center, a 10.13039/100000874Brain and Behavior Research Foundation independent investigator award (D.K.), a 10.13039/100007631Canadian Institute for Advanced Research fellowship (D.K.), a 10.13039/100000001National Science Foundation Graduate Research Fellowship (K.L.), and an 10.13039/100005280American Association of University Women American Dissertation Fellowship (K.L.).

## Declaration of competing interest

The authors declare that they have no known competing financial interests or personal relationships that could have appeared to influence the work reported in this paper.

## Data Availability

Raw sequencing data has been uploaded to the NCBI Gene Expression Omnibus (Submission number GSE297030) and is publicly available from the date of publication. All other data associated with this study are available upon request.

## Appendix A Supplementary data

S1 Statistics S1 Statistics

S2 Supplementary data for gene comparisons S2 Supplementary data for gene comparisons
