# Supplementary material for: Transcriptomic profiles of susceptibility and resilience to stress in the amygdala and hippocampus of male rats
Source: Neurobiol Stress. 2025 Aug 26;38:100754. doi: 10.1016/j.ynstr.2025.100754 (PMC12445234; doi:10.1016/j.ynstr.2025.100754)
Supplement: S1 Statistics [file mmc1.pdf]

Figure	Panel	Dependent Variable	Transformation applied	Test	n	Independent variable(s)	Test Statistic (degrees of freedom)	p value	Notes: Violations of normality, equality of variance, etc.
1	B	% weight change	None	Welch's t test	Control n = 14<br>Stress n = 16	Stress	t(24.38) = 7.466	< 0.0001	A Welch's t test was used due to unequal variance (F = 3.059, p = 0.0496). Some animals are missing from this analysis due to experimental error of not weighing on the day of stress.
	C	Corticosterone	n/a	No comparisons made	n/a	n/a	n/a	n/a	
	D	Anxiety score	n/a	No comparisons made	n/a	n/a	n/a	n/a	
	E	Composite anxiety-like behavior score	None	Generalized linear model with Poisson distribution	Control n = 19<br>Stress n = 20	Stress	Estimate of coefficient: 0.41087 +/- 0.14274	0.00399597	
	F	Composite avoidance score	None	Generalized linear model with Poisson distribution	Control n = 19<br>Stress n = 20	Stress	Estimate of coefficient: 0.26360 +/- 0.08064	0.00108036	
		Composite startle score	None	Generalized linear model with Poisson distribution	Control n = 19<br>Stress n = 20	Stress	Estimate of coefficient: -0.05129 +/- 0.31623	0.87114549	
	G	Correlation of % weight change vs. composite anxiety-like behavior score in stress-exposed animals	None	Pearson correlation	n = 16	n/a	r = -0.468	0.068	Some animals are missing from this analysis due to experimental error of not weighing on the day of stress.
	H	Correlation of corticosterone at 30' into stress vs. composite anxiety-like behavior score in stress-exposed animals	None	Pearson correlation	n = 18	n/a	r = 0.2443	0.3287	2 serum samples were not run due to limited assay reagent
	I	Correlation of corticosterone at 3 hours into stress vs. composite anxiety-like behavior score in stress-exposed animals	None	Pearson correlation	n = 18	n/a	r = 0.5512	0.0177	2 serum samples were not run due to limited assay reagent
S2		OFT(L) time in center	None	Unpaired t test	Control n = 19<br>Stress n = 20	Stress	t(37) = 0.9476	0.3495	
		OFT(D) time in center	None	Mann-Whitney test	Control n = 19<br>Stress n = 20	Stress	U = 177	0.7284	Failed D'Agostino-Pearson omnibus (K2) test before and after log transformation
		EPM(L) time in open	None	Unpaired t test	Control n = 19<br>Stress n = 20	Stress	t(37) = 1.412	0.1663	
		EPM(D) time in open	None	Unpaired t test	Control n = 19<br>Stress n = 20	Stress	t(37) = 1.564	0.1262	
		LD time in open	None	Mann-Whitney test	Control n = 19<br>Stress n = 20	Stress	U = 158.5	0.3827	Failed D'Agostino-Pearson omnibus (K2) test before and after log transformation
		OFT(L) frequency in center	None	Generalized linear model with Poisson distribution	Control n = 19<br>Stress n = 20	Stress	Estimate of coefficient: 0.0031 +/- 0.0825	0.96962425	
		OFT(D) frequency in center	None	Generalized linear model with Poisson distribution	Control n = 19<br>Stress n = 20	Stress	Estimate of coefficient: 0.1721 +/- 0.0857	0.0446	
		EPM(L) latency to open	Log(Y)	Unpaired t test	Control n = 19<br>Stress n = 20	Stress	t(37) = 0.4274	0.6716	Failed D'Agostino-Pearson omnibus (K2) test. Passed after log transformation.
		EPM(D) latency to open	None	Mann-Whitney test	Control n = 19<br>Stress n = 20	Stress	U = 130	0.0932	Failed D'Agostino-Pearson omnibus (K2) test before and after log transformation
		LD frequency in open	None	Generalized linear model with Poisson distribution	Control n = 19<br>Stress n = 20	Stress	Estimate of coefficient: 0.2440 +/- 0.1510	0.1062	
		OFT(L) latency to center	Log(Y+1)	Unpaired t test	Control n = 19<br>Stress n = 20	Stress	t(37) = 0.0002	0.9998	Failed D'Agostino-Pearson omnibus (K2) test. Passed after log transformation.
		OFT(D) latency to center	Log(Y+1)	Unpaired t test	Control n = 19<br>Stress n = 20	Stress	t(37) = 1.458	0.1533	Failed D'Agostino-Pearson omnibus (K2) test. Passed after log transformation.
		EPM(L) time in closed	None	Unpaired t test	Control n = 19<br>Stress n = 20	Stress	t(37) = 1.799	0.0801	
		EPM(D) Time in closed	None	Unpaired t test	Control n = 19<br>Stress n = 20	Stress	t(37) = 2.204	0.0338	
		LD latency to open	Log(Y)	Unpaired t test	Control n = 19<br>Stress n = 20	Stress	t(37) = 1.239	0.2231	Failed D'Agostino-Pearson omnibus (K2) test. Passed after log transformation.
		ASR Habituation phase startle	None	Two way repeated measures ANOVA	Control n = 19<br>Stress n = 20	Stress x Time	F (1, 37) = 1.009	0.3216	
						Stress	F (1, 37) = 0.1556	0.6955	
						Time	F (1, 37) = 92.05	<0.0001	
						Subject	F (37, 37) = 0.6568	0.8971	
		ASR Habituation phase % habituation	None	Mann-Whitney test	Control n = 19<br>Stress n = 20	Stress	U = 157	0.3653	Failed D'Agostino-Pearson omnibus (K2) test before and after log transformation
		ASR Habituation phase mean startle	None	Unpaired t test	Control n = 19<br>Stress n = 20	Stress	t(37) = 0.4939	0.6243	
		ASR Threshold phase startle	None	Two way repeated measures ANOVA	Control n = 19<br>Stress n = 20	Stimulus x Stress	F (4, 148) = 0.5143	0.7253	
						Stimulus	F (4, 148) = 88.13	<0.0001	
						Stress	F (1, 37) = 0.006780	0.9348	
						Subject	F (37, 148) = 11.38	<0.0001	
		ASR Threshold phase startle curve AUC	None	Unpaired t test	Control n = 19<br>Stress n = 20	Stress	t(37) = 0.6505	0.5194	
S4	A	Correlation of % weight change vs. composite anxiety-like behavior score in stress-exposed animals	None	Pearson correlation	n = 16	n/a	r = -0.468	0.068	Some animals are missing from this analysis due to experimental error of not weighing on the day of stress.
		Correlation of % weight change vs. composite avoidance behavior score in stress-exposed animals	None	Pearson correlation	n = 16	n/a	r = -0.3477	0.187	Some animals are missing from this analysis due to experimental error of not weighing on the day of stress.
		Correlation of % weight change vs. composite startle behavior score in stress-exposed animals	None	Pearson correlation	n = 16	n/a	r = -0.605	0.013	Some animals are missing from this analysis due to experimental error of not weighing on the day of stress.
	B	Correlation of corticosterone at 30' into stress vs. composite anxiety-like behavior score in stress-exposed animals	None	Pearson correlation	n = 18	n/a	r = 0.2443	0.3287	2 serum samples were not run due to limited assay reagent
		Correlation of corticosterone at 30' into stress vs. composite avoidance behavior score in stress-exposed animals	None	Pearson correlation	n = 18	n/a	r = 0.2263	0.3665	2 serum samples were not run due to limited assay reagent
		Correlation of corticosterone at 30' into stress vs. composite startle behavior score in stress-exposed animals	None	Pearson correlation	n = 18	n/a	r = 0.1332	0.5982	2 serum samples were not run due to limited assay reagent
	C	Correlation of corticosterone at 3 hours into stress vs. composite anxiety-like behavior score in stress-exposed animals	None	Pearson correlation	n = 18	n/a	0.5512	0.0177	2 serum samples were not run due to limited assay reagent
		Correlation of corticosterone at 3 hours into stress vs. composite avoidance behavior score in stress-exposed animals	None	Pearson correlation	n = 18	n/a	0.5039	0.033	2 serum samples were not run due to limited assay reagent
		Correlation of corticosterone at 3 hours into stress vs. composite startle behavior score in stress-exposed animals	None	Pearson correlation	n = 18	n/a	0.3275	0.1846	2 serum samples were not run due to limited assay reagent
S6	A	Correlation of Cartpt vs. composite anxiety-like behavior score in stress-exposed animals	None	Pearson correlation	n = 11	n/a	r = 0.8089	0.0026	
	B	Correlation of Cartpt vs. composite avoidance behavior score in stress-exposed animals	None	Pearson correlation	n = 11	n/a	r = 0.7993	0.0032	
	C	Correlation of Cartpt vs. composite startle behavior score in stress-exposed animals	None	Pearson correlation	n = 11	n/a	r = 0.3941	0.2304	
