# Supplementary material for: Transcriptomic profiles of susceptibility and resilience to stress in the amygdala and hippocampus of male rats
Source: Neurobiol Stress. 2025 Aug 26;38:100754. doi: 10.1016/j.ynstr.2025.100754 (PMC12445234; doi:10.1016/j.ynstr.2025.100754)
Supplement: S3 Supplemental Figures [file mmc3.docx]

**Supplementary Materials**

**Transcriptomic profiles of susceptibility and resilience to stress in the amygdala and hippocampus of male rats**

Kimberly L. P. Long^1,^*^,Ѱ^, Sandra E. Muroy^2,^*, Siamak K. Sorooshyari^2,#^, Mee Jung Ko^1^, Yanabah Jaques^1^, Kishant Mohan^3^, Peter Sudmant^2^, Daniela Kaufer^1,2,†^

^1^Helen Wills Neuroscience Institute, University of California, Berkeley, Berkeley, CA 94720, USA.

^2^Department of Integrative Biology, University of California, Berkeley, Berkeley, CA 94720, USA.

^3^Department of Bioengineering, University of California, Berkeley, Berkeley, CA 94720, USA.

*These authors contributed equally to this work.

^†^To whom correspondence should be addressed: danielak@berkeley.edu, (510) 642-9346

^Ѱ^Current address: Department of Psychiatry and Behavioral Sciences, University of California, San Francisco, San Francisco, CA 94143, USA.

^#^Current address: Department of Statistics, Stanford University, Stanford, CA 94305, USA.


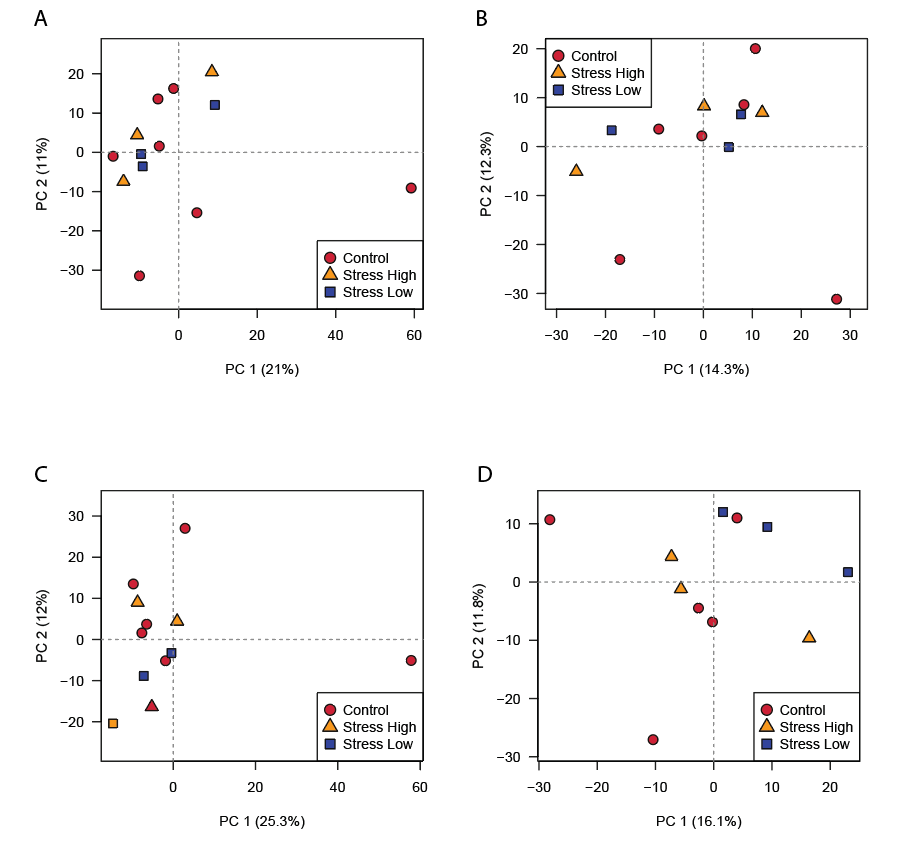


**Supplemental Figure 1:** PCA of normalized gene expression values from basolateral amygdala (BLA) and hippocampal dentate gyrus (DG) samples. A. BLA with all samples included. B. BLA with outlier control sample removed. C. DG with all samples. D. DG with outlier control sample removed.


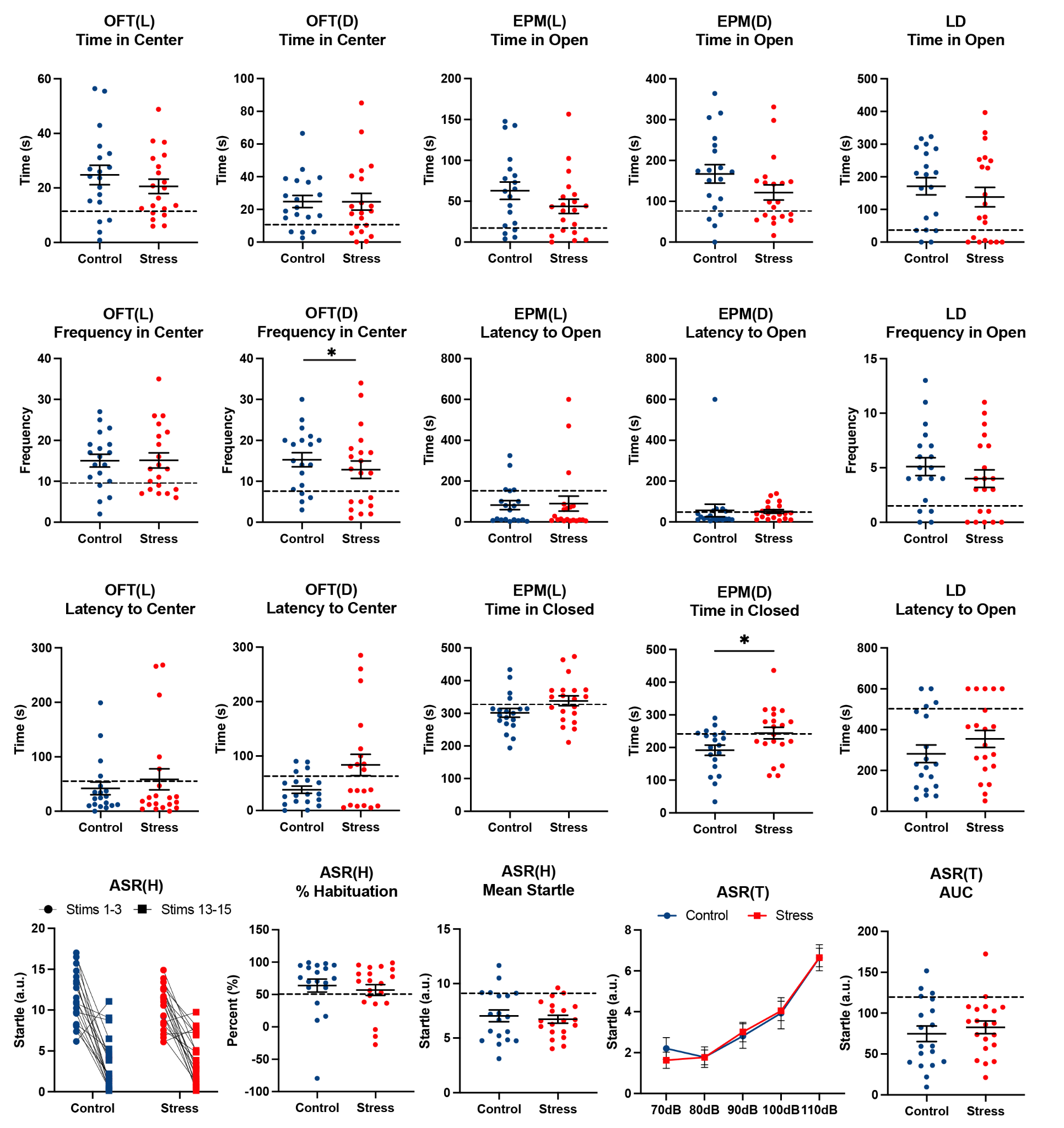


**Supplemental Figure 2:** All behavior data. Dotted line marks the 20^th^ percentile of the control distribution. OFT(L), open field test under bright lights; OFT(D), open field test under dim lights; EPM(L), elevated plus maze under bright lights; EPM(D), elevated plus maze under dim red lights; LD, light-dark box; ASR(H) 1-3, acoustic startle response test habituation phase, mean startle to stimuli 1-3; ASR(H) 13-15, ASR(H) mean startle to stimuli 13-15; ASR % Habituation, ASR(H) percent habituation; ASR(H), Mean all, mean startle response to all 15 stimuli; ASR(T) AUC, area under the curve from the ASR threshold phase. Statistics: OFT(D) Frequency in center: Poisson generalized linear model, coefficient 0.172 +/- 0.086, p = 0.045; EPM(D) Time in closed arm: t(37) = 2.204, p = 0.034. *p < 0.05.


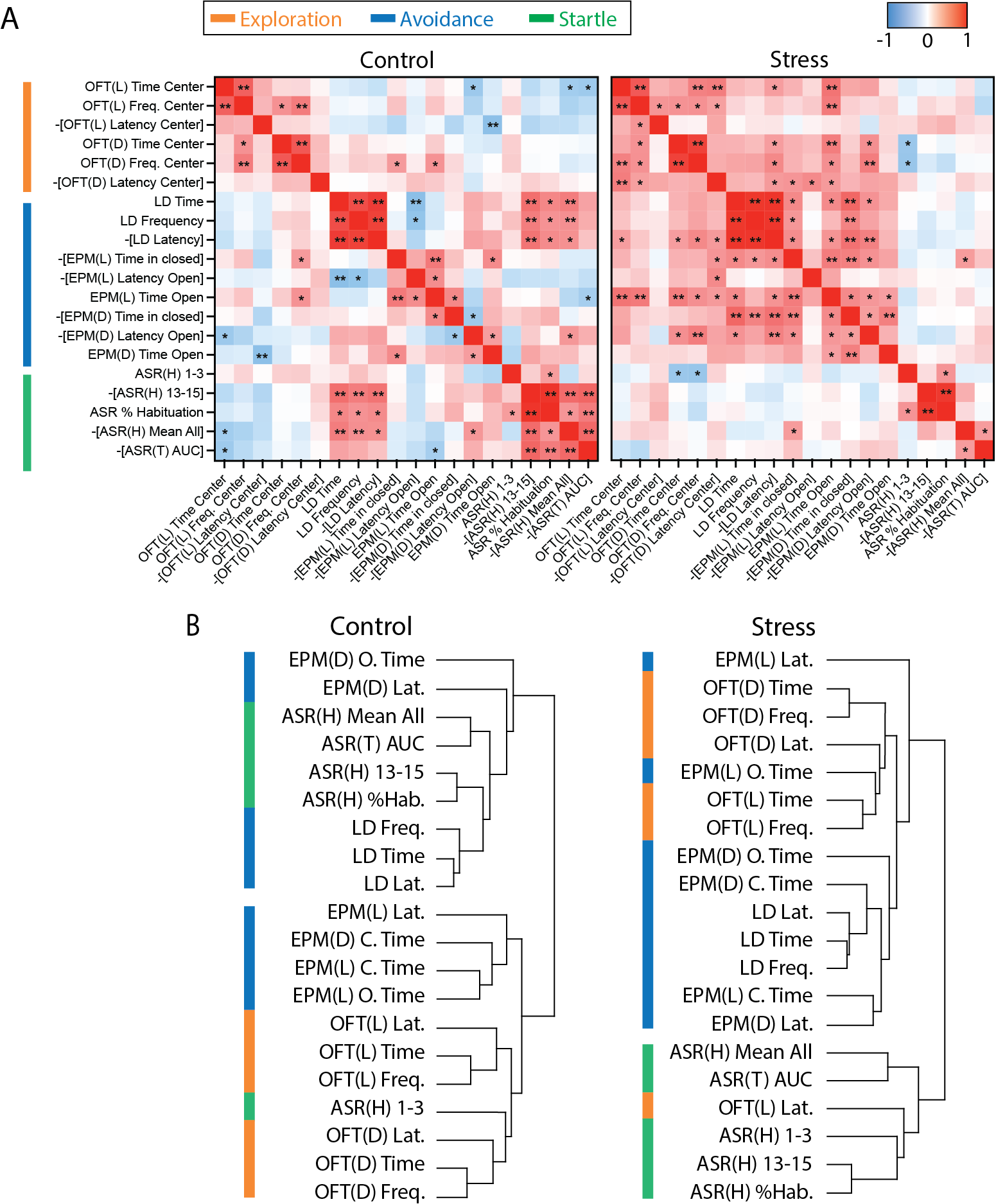


**Supplemental Figure 3:** Behavioral correlations and clustering. A. Heatmaps of correlations between behavioral metrics in control (left) and stress-exposed (right) rats. Pearson r values range from blue (-1) to red (+1), and asterisks within squares mark significant correlations. Colored bars classify individual behaviors according to their hypothesized representation in the literature (for example, elevated plus maze is typically used to measure anxiety-like avoidance behavior in rodents). For clarity of presentation, behavioral metrics with opposite valence (for example, time spent in the closed arm of the elevated plus maze) from those of standard metrics (for example, time spent in the open arms of the elevated plus maze) were multiplied by -1. B. Hierarchical clustering of behavioral metrics in control (left) and stress-exposed (right) rats. OFT(L), open field test under bright lights; OFT(D), open field test under dim lights; EPM(L), elevated plus maze under bright lights; EPM(D), elevated plus maze under dim red lights; LD, light-dark box; ASR(H) 1-3, acoustic startle response test habituation phase, mean startle to stimuli 1-3; ASR(H) 13-15, ASR(H) mean startle to stimuli 13-15; ASR % Habituation, ASR(H) percent habituation; ASR(H), Mean all, mean startle response to all 15 stimuli; ASR(T) AUC, area under the curve from the ASR threshold phase. *p < 0.05, **p < 0.01


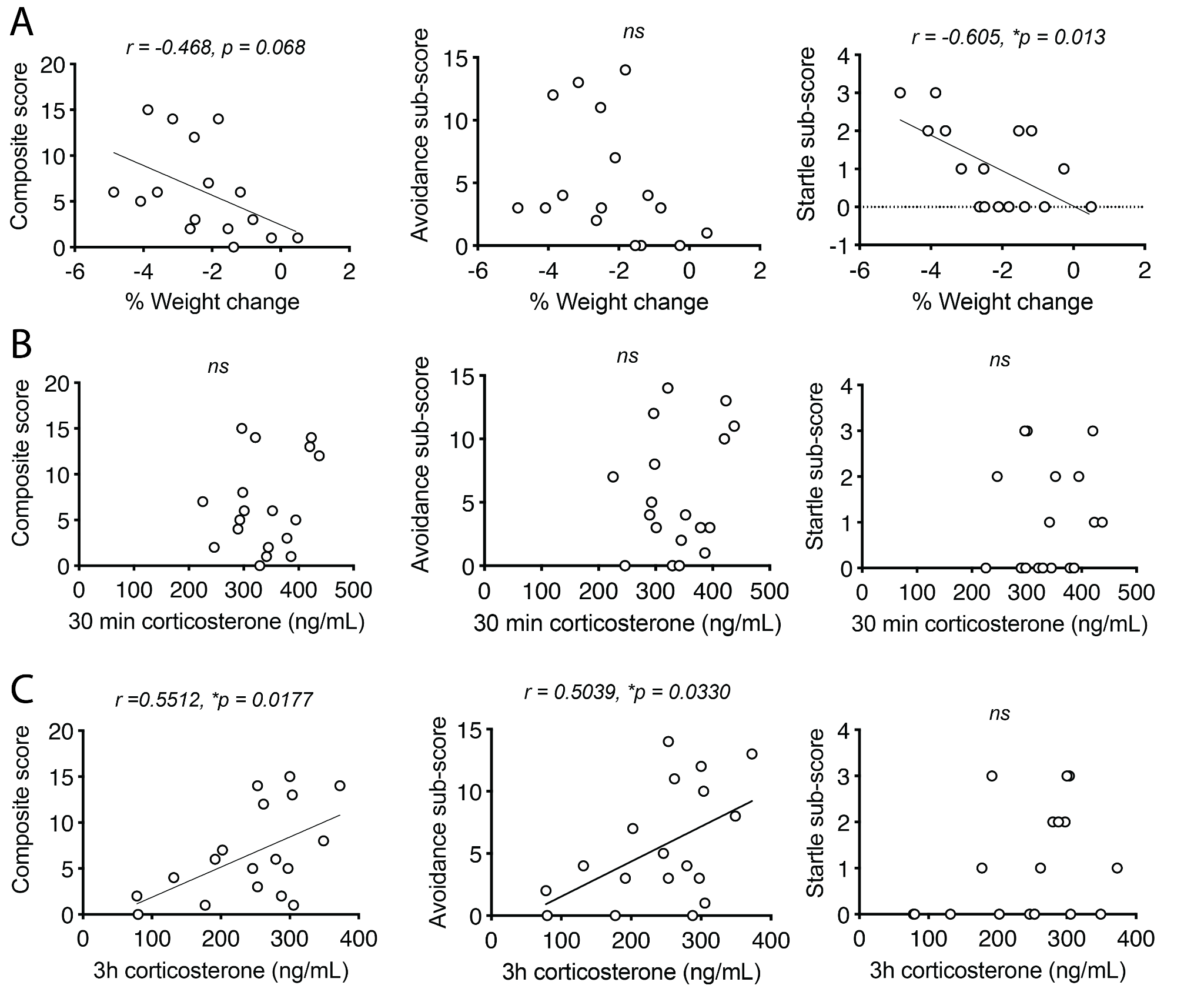


**Supplemental Figure 4:** Correlations between corticosterone, weight loss, and behavior after stress. A. Correlations between percent weight change from day of stress to day after stress and composite behavior scores (left, overall score: r = -0.468, p = 0.068; middle, avoidance sub-score: r = -0.3477, p = 0.187; right, startle sub-score: r = -0.605, p = 0.013). B. Correlations between serum corticosterone at 30 minutes and composite behavior scores (left, overall score: r = 0.2443, p = 0.3287; middle, avoidance sub-score: r = 0.2263, p = 0.3665; right, startle sub-score: r = 0.1332, p = 0.5982). C. Correlations between serum corticosterone at 3 hours and composite behavior scores (left, overall score: r = 0.5512, p = 0.0177; middle, avoidance sub-score: r = 0.5039, p = 0.033; right, startle sub-score: r = 0.3275, p = 0.1846). *p < 0.05


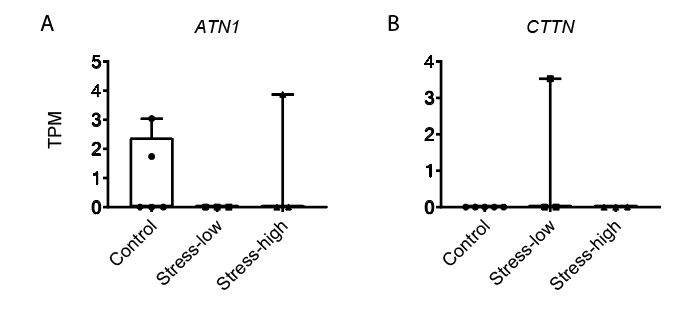


**Supplemental Figure 5:** *ATN1* and *CTTN* gene expression differences between control, stress-low, and stress-high groups. A. *ATN1* gene expression (in transcripts per million [TPM]) for the 3 groups. B. *CTTN* gene expression (in TPM) for the 3 groups. Note that in both cases, outlier values drive group differences.


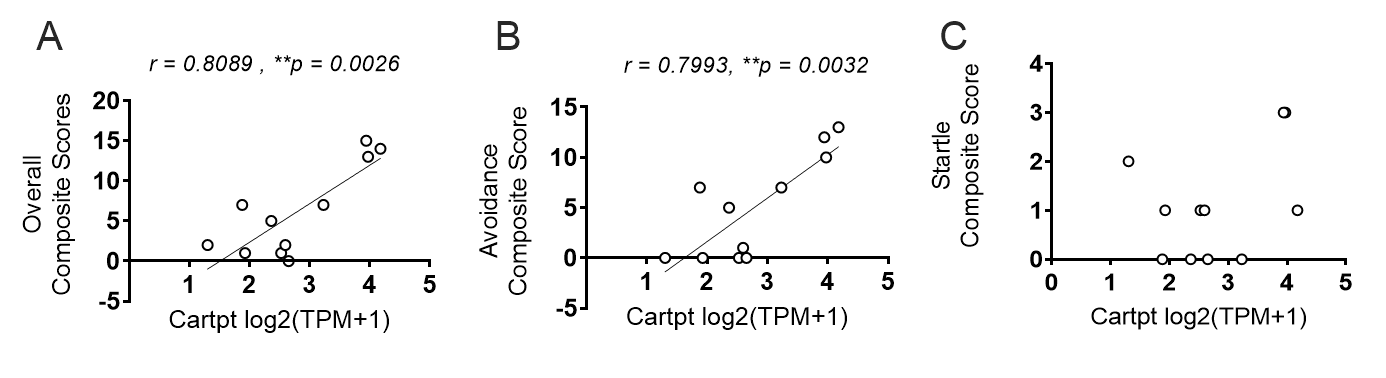


**Supplemental Figure 6:** *Cartpt* gene expression correlations to overall, avoidance, and startle composite scores. A. Pearson correlation of *Cartpt* gene expression to overall composite scores (r = 0.8089, p = 0.0026). B. Correlation of *Cartpt* gene expression to avoidance composite sub-scores (r = 0.7993, p = 0.0032). C. Correlation of *Cartpt* gene expression to startle composite sub-scores (r = 0.3941, p = 0.2304).

**Supplemental file S1** “Statistics”

**Supplemental file S2** “Supplementary data for gene comparisons”
